# A Novel Pyrimidin-Like Plant Activator Stimulates Plant Disease Resistance and Promotes Growth

**DOI:** 10.1371/journal.pone.0123227

**Published:** 2015-04-07

**Authors:** Tie-Jun Sun, Yun Lu, Mari Narusaka, Chao Shi, Yu-Bing Yang, Jian-Xin Wu, Hong-Yun Zeng, Yoshihiro Narusaka, Nan Yao

**Affiliations:** 1 State Key Laboratory of Biocontrol, Guangdong Key Laboratory of Plant Resources, School of Life Sciences, Sun Yat-sen University, Guangzhou, P. R. China; 2 Research Institute for Biological Sciences Okayama, Okayama, Japan; Key Laboratory of Horticultural Plant Biology (MOE), CHINA

## Abstract

Plant activators are chemicals that induce plant defense responses to a broad spectrum of pathogens. Here, we identified a new potential plant activator, 5-(cyclopropylmethyl)-6-methyl-2-(2-pyridyl)pyrimidin-4-ol, named PPA (pyrimidin-type plant activator). Compared with benzothiadiazole S-methyl ester (BTH), a functional analog of salicylic acid (SA), PPA was fully soluble in water and increased fresh weight of rice (*Oryza sativa*) and *Arabidopsis* plants at low concentrations. In addition, PPA also promoted lateral root development. Microarray data and real-time PCR revealed that PPA-treated leaves not challenged with pathogen showed up-regulation of genes related to reactive oxygen species (ROS), defenses and SA. During bacterial infection, *Arabidopsis* plants pretreated with PPA showed dramatically decreased disease symptoms and an earlier and stronger ROS burst, compared with plants pretreated with BTH. Microscopy revealed that H_2_O_2_ accumulated in the cytosol, plasma membrane and cell wall around intracellular bacteria, and also on the bacterial cell wall, indicating that H_2_O_2_ was directly involved in killing bacteria. The increase in ROS-related gene expression also supported this observation. Our results indicate that PPA enhances plant defenses against pathogen invasion through the plant redox system, and as a water-soluble compound that can promote plant growth, has broad potential applications in agriculture.

## Introduction

In their natural environments, plants encounter a large variety of pathogens, including fungi, oomycetes, viruses, bacteria, and nematodes [1]. Plant defenses include pathogen-associated molecular pattern (PAMP)-triggered immunity (PTI) [2–3], and effector-triggered immunity (ETI) [4–5]. PTI induction involves MAP kinase signaling pathways, transcriptional induction of pathogenesis-related (*PR*) genes, a burst of reactive oxidative species (ROS), phytoalexin production and deposition of callose to limit pathogen infection and growth [6]. However, pathogens have evolved mechanisms to suppress PTI by secreting effectors into the apoplast or directly into the cytoplasm. These effectors presumably alter resistance signaling or manifestation of resistance responses, often by mimicking or inhibiting eukaryotic cellular functions [5]. To counteract pathogen effectors, plants have developed a mechanism using resistance (R) proteins to recognize different pathogen effectors directly or indirectly, and elicit ETI [4]. ETI occurs as an accelerated and amplified PTI response, often associated with a rapid, hypersensitive cell death response (HR) at the infection site [5].

In addition to the local responses of PTI and ETI, pathogens can also induce a long-lasting defense response in plants, called systemic acquired resistance (SAR), characterized by the local production of signals such as SA, methyl salicylic acid, azelaic acid, glycerol-3-phosphate, and abietanediterpenoid dehydroabietinal [7–11] and the rapid translocation of these signals to undamaged tissues [11–13]. These signals then lead to the systemic expression of *PR* genes, phytoalexin accumulation and cell wall strengthening in undamaged distal tissue to protect the rest of the plant from secondary invasion [12]. In contrast to ETI, SAR does not involve coupled HR, but instead promotes cell survival. Recent reports found that SAR also has *trans*-generational benefits, where immune ‘memory’ can pass to the next generation [13–14]. Despite intense research, our understanding of SAR signaling pathways remains obscure.

Plant defense responses can also be activated by application of SA, or its synthetic analogs 2,6-dichloroisonicotinic acid (INA) and benzothiadiazole S-methyl ester (BTH) [12,15]. These chemicals are called plant activators. In *Arabidopsis thaliana*, BTH treatment induced accumulation of *PR1* mRNA at 4 h after treatment and *PR1* mRNA increased 24 h and 48 h, and then decreased after 96 h [15]. BTH treatment protected wheat fields from powdery mildew and cauliflower from downy mildew of crucifers caused by *Peronospora parasitica* [16–17]. In addition to their ability to induce defenses, these plant activators also are derived from plant metabolic products, usually do not kill pathogens directly, have low molecular weights and produce little or no pollution. Based on these key differences from traditional pesticides, plant activators may be more suitable for pathogen control in agricultural systems.

Up to now, few synthetic compounds with high SAR activity have been reported, and the agricultural applications of plant SAR activators remain far from developed [18–19]. BTH is the most successful commercial plant SAR activator but its obvious shortcomings limit its application in crop production. For example, Canet et al. (2010) [20] reported that BTH-treated plants have less biomass than mock-treated plants without pathogen inoculation, even with low concentrations of BTH. This observation reveals the cost of fitness and resistance in the absence of pathogen [21, 22]. Because BTH cannot dissolve in water, it may also cause some secondary pollution from the organic solvent.

One of the earliest cellular responses to pathogen attack is ROS production. Superoxide (O_2_^-^) or its dismutation product hydrogen peroxide (H_2_O_2_) are generated in the apoplast from two (or more) different sources at the plant cell surface: cell wall peroxidases *PEROXIDASE33* (*PRX33*), *PRX34* and NADPH oxidases, known as respiratory burst oxidase homologues (*RbohD*, *RbohF*) [23,24]. In plant cells, ROS can directly strengthen cell walls by cross-linking glycoproteins to resist pathogen invasion and also have direct effects on pathogens [24,25]. Moreover, ROS mediate vital plant defense responses and signal cascades [23,26,27]. Reduction of ROS scavenging systems can increase ROS levels and activation resistance after infection [28]. Collectively, ROS production and scavenging systems could both contribute to fine-tuning ROS levels and signaling pathways in the response to pathogen attack [24].

In this study, we report a new pyrimidin-type plant activator (PPA). Its water solubility and effects on plant development and root system were tested and compared with BTH. Unlike BTH, PPA promoted plant biomass increase and root development. ROS, defense- and SA related-genes were elevated after application of PPA. We found that PPA induced immune responses against pathogen infection. We propose that PPA induces plant defense programs by moderating ROS and may be suitable for agricultural applications due to its effects on plant growth and defenses.

## Materials and Methods

### Materials

*Arabidopsis thaliana* wild-type plants (Col-0) were grown on soil in the greenhouse or sown on 1/2x Murashige Skoog (MS) medium supplemented with the indicated chemicals under 16 h light /8 h dark, as described previously [29]. Rice plants (*Oryza sativa ssp*. *japonica* c.v. Nipponbare) were grown in water or indicated chemicals and incubated at room temperature. Benzothiadiazole S-methyl ester (BTH) and 5-(cyclopropylmethyl)-6-methyl-2-(2-pyridyl) pyrimidin-4-ol (PPA) were purchased from WAKO (Japan) and Maybridge (United Kingdom), respectively. Trypan blue, diaminobenzidine tetrahydrochloride (DAB) and cerium chloride were purchased from Sigma. *Pseudomonas syringae* pv. *maculicola* strain DG3 (virulent) was kindly obtained from Dr. Jean Greenberg and inoculated as described previously [30].

### *In vitro* effect on pathogen after PPA treatments

*P*. *syringae* pv. *maculicola* (strain DG3) was grown in King’s B Medium and treated with 300 μM BTH and 40 μM PPA. BTH was dissolved in acetone (the final acetone concentration was never higher than 0.3%). The OD600 was recorded every two hours. *Botrytis cinerea* strain NJ-09 was cultured on Potato Dextrose Agar (PDA) medium and spores were collected. The spores were germinated on glass slides covered with 1% agar containing 300 μM BTH and 40 μM PPA, and then germination rates were counted under a microscope (Axio Imager A1, Carl Zeiss) after 12 h treatments.

### Trypan blue and DAB staining

Leaves were sampled and boiled in lactophenol solution (lactic acid: glycerol: liquid phenol: distilled water = 1:1:1:1) containing 0.025% trypan blue for 30 sec, and then boiled in 95% ethanol:lacophenol (2:1) for 1 min. Leaves were transferred to 50% ethanol for washing, kept in distilled water and observed under a microscope (Axio Imager A1, Carl Zeiss). For DAB staining, samples were immersed in 1 mg/mL DAB (pH 5.5) for 2 h, boiled in 95% ethanol for 2 min, then washed in 50% ethanol and kept in distilled water at 4°C. Photographs were taken with a stereomicroscope (SteREO Lumar.V12, Carl Zeiss).

### H_2_O_2_ detection by CeCl_3_ staining

The histochemical cerium chloride method was used to detect H_2_O_2_ based on generation of cerium hydroxide, as described previously [31]. The leaves were cut and incubated in 10 mM CeCl_3_ dissolved in 50 mM MOPS buffer (pH 7.2) for 1 h. Control samples were incubated in MOPS buffer only. Samples were fixed in 2.5% (v/v) glutaraldehyde and 2% (v/v) paraformaldehyde in 0.1 M cacodylate buffer (pH 7.2–7.4). Samples were embedded in EPON-812 medium. Ultrathin sections were obtained on a microtome (Leica EM UC6, Vienna, Austria) and examined without staining. The images were photographed using a transmission electron microscope (JEM-1400, JEOL, Tokyo, Japan) at an accelerating voltage of 120 kV.

### Gene expression and microarray data analysis

For gene expression analysis, real-time quantitative RT-PCR was performed as described previously [29,41]. Briefly, total RNA was extracted with the EZNA Plant RNA Kit (Omega Bio-Tek), and reverse transcribed to cDNA using the PrimeScript RT reagent Kit with gDNA Eraser (Takara). The PCR efficiency of target gene and internal control gene *ACT2* (At3g18780) were determined and adjusted to similar values. The cDNA was quantified using gene-specific primers and the SYBR Premix Ex Taq II reagent in a StepOne Plus (Applied Biosystems). Three technical replicates were performed for each template and primer combination. The 2^-ΔΔCT^ method [32,41] was used to calculate the relative expression level of target genes according to the expression level of *ACT2*. The primers for amplification are listed in S3 Table. Each experiment was repeated three times.

The RNA samples were extracted after treatments and then sent to TAKARA BIO INC (http://www.takara-bio.com/index.htm) for microarray analysis with the Affymetrix *Arabidopsis* ATH1 GeneChip arrays (Affymetrix, http://www.affymetrix.com). Raw data were then processed using Affymetrix software, including AGCC (Affymetrix GeneChip Command Console Software), Expression Console (Affymetrix Expression console software) and other tools such as GeneSpring (Agilent Technologies, http://www.agilent.com). After preliminary processing, three sets of differentially-expressed genes were identified by comparing 5 h, 10 h and 24 h data with 0 h data. The expressed genes were annotated by referring to the TAIR database (http://www.arabidopsis.org/), genes not annotated in TAIR were annotated by referring to the Affymetrix official annotation of GPL198 platform, and genes related to "defense", "salicylic acid" and "ROS" were picked based on their GO (Gene Ontology) terms.

### Statistical analysis

Statistical analyses were performed with Fisher’s protected least significant difference (PLSD), a post-hoc multiple comparison tests (Statview statistical package 5.0.1) or Student's *t*-test. Statistical significance was considered when *P<*0.05. Data are presented as means ± standard deviation. All experiments were repeated at least three times with similar results.

## Results

### Characteristics of the plant activator PPA

**Fig 1:**
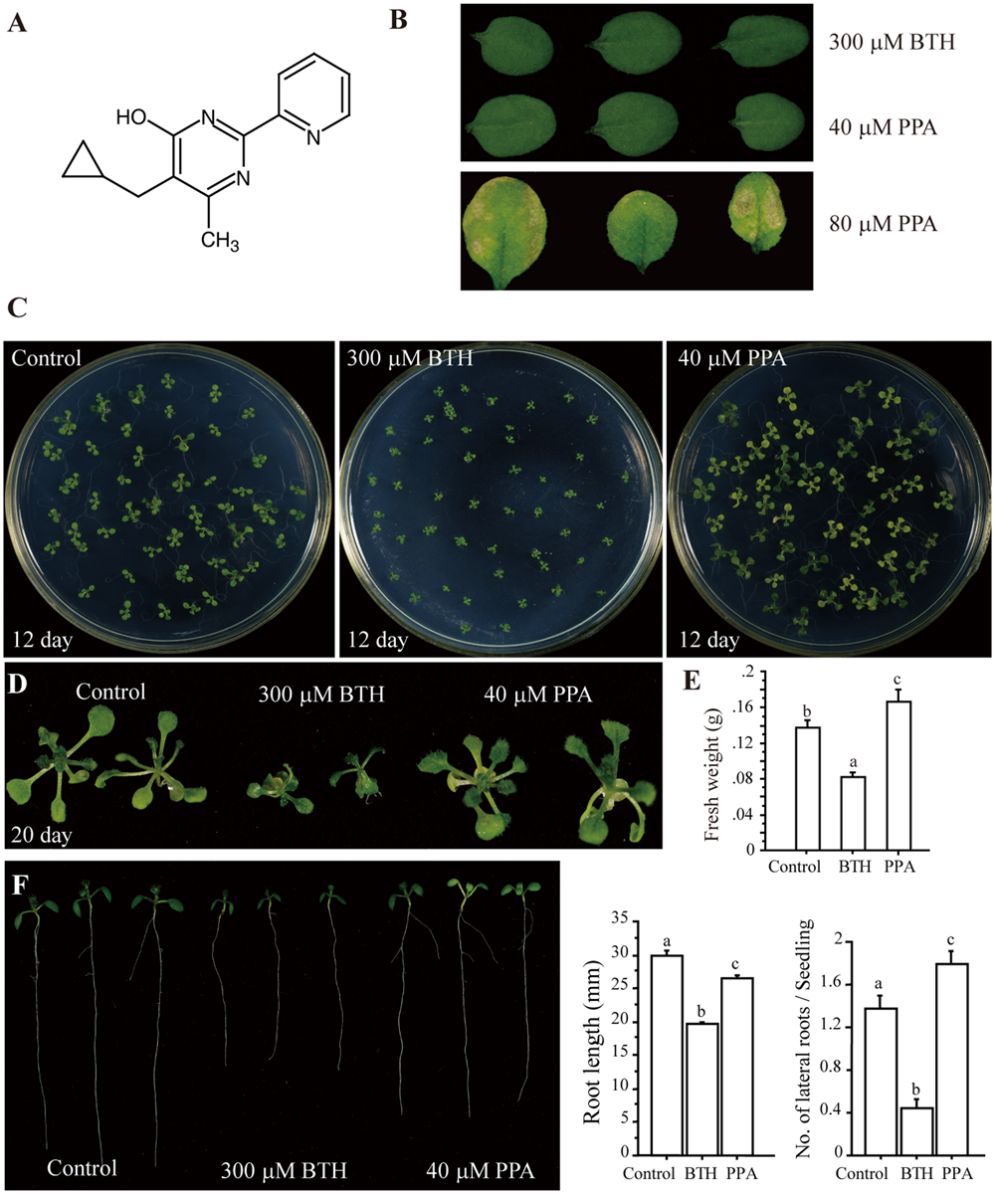
A plant activator and its effect on *Arabidopsis* development. **A,** The structure of pyrimidin-type plant activator (PPA). **B**, Determination of a suitable application concentration of PPA. Plants were sprayed with 300 μM BTH (dissolved in 0.3% acetone) or the indicated concentration of PPA (dissolved in distilled water) and 9 days later observed. **C** and **D,** Phenotypes of *Arabidopsis* seedlings grown on 1/2x Murashige and Skoog (MS) plates containing BTH or PPA for 12 days (**C**) and 20 days (**D**). **E,** Comparison of plant biomass with different treatments. Eighteen-day-old plants were treated with 300 μM BTH or 40 μM PPA twice. Twelve days later the fresh weight was measured. **F,** Effect of plant activators on *Arabidopsis* root development. Seeds were sown on 1/2x MS horizontal plates and grown for 4 days. Roots of similar lengths were selected and transferred to a 1/2x MS vertical plate containing 300 μM BTH, 40 μM PPA or 0.3% acetone (control). Photos were taken after 5 days. At least fifty independent samples were used for statistical analysis of the length of primary roots (middle panel) and the number of lateral roots per seedling (right panel). Data sets marked with letters indicate significant differences (P<0.05, PLSD-test). The values shown are the averages of three independent experiments. This experiment was repeated three times with similar results, using independent samples.

Plant activators include natural or synthetic compounds that stimulate plant defense responses, providing protection against a wide spectrum of plant pathogens [19]. C_14_H_15_N_3_O, a pyrimidin-type plant activator (PPA), is a water soluble white powder and its chemical structure has no distinct similarity to other plant activators such as BTH and INA (Fig 1A and S1A Fig). Moreover, except β-aminobutryric acid (BABA), other well-known plant activators, such as BTH, do not dissolve in water. To examine the effect of PPA on plant phenotype, we treated plants with different concentrations of PPA and compared them to plants treated with 300 μM BTH, a standard concentration used in previous reports [15,17]. We found that 40 μM PPA produced a response similar to 300 μM BTH (Fig 1B), but without any harmful phenotype when sprayed on plants; therefore, we used this concentration for further studies.

To verify whether PPA induced macroscopic cell death, we used trypan blue staining to examine cell death and DAB staining to examine the ROS burst. We observed no visible cell death (S1B Fig) or DAB precipitation (S1C Fig) in 3-week-old plants treated with 300 μM BTH or 40 μM PPA for 9 days, indicating that this concentration of PPA does not harm plants.

### Effect of PPA on plant biomass and roots

Previous reports indicated that BTH treatment causes a dose-dependent decrease in plant biomass [16,20,21]. During our experiments, we found that BTH-treated seedlings and plants have less biomass than mock-treated plants, for both *Arabidopsis* (Fig 1C–1E and S1D Fig) and rice plants (S1E Fig), but PPA-treated plants showed no adverse effect on biomass, when grown on 1/2x MS plates containing 40 μM PPA (Fig 1C–1E and S1D Fig).

We further investigated the effect of PPA on seedling root development. The root system comprises a primary root and lateral roots and functions in the uptake of nutrients and water and in the physical anchoring of plants [33]. Previous BTH studies did not report an effect of BTH on plant root development. We found that BTH-treated plants had significantly shorter primary roots, compared with PPA and mock-treated groups (Fig 1F). Also, the PPA-treated seedlings had more lateral roots than control seedlings. These results indicated that PPA slightly affects the length of Arabidopsis primary roots, but promotes the number of lateral roots and increases plant biomass.

### Identification of gene expression changes induced by PPA treatment

**Fig 2:**
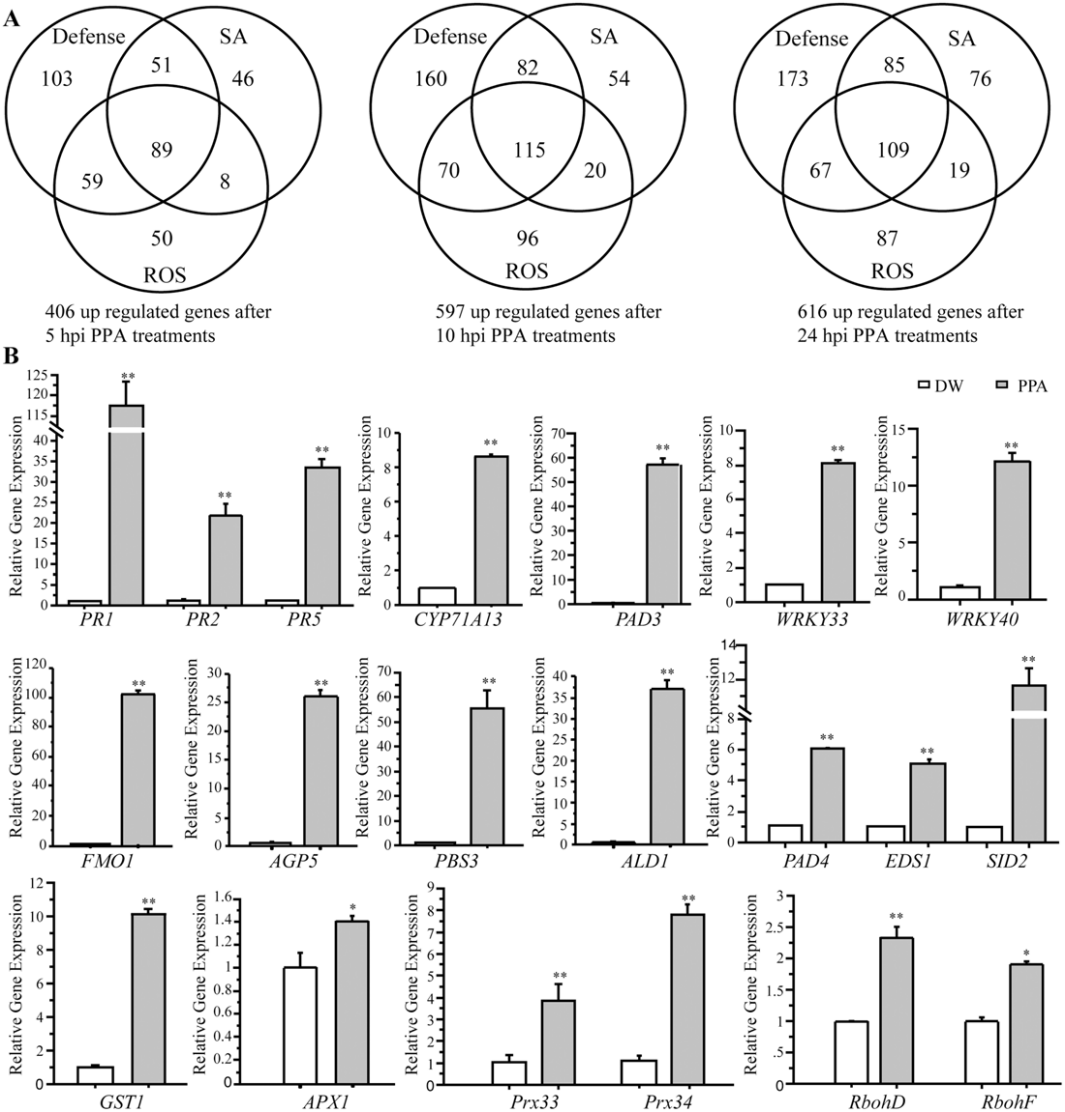
Induction of gene expression by PPA treatment. **A**, 20-day-old plants were treated with 80 μM PPA and tissues were harvested at 0 h, 5 h, 10 h, 24 h. Using ANOVA (P<0.05) and a 2-fold change cutoff (Log_2_≥1), we identified genes with altered expression. The Venn diagram shows clusters of up-regulated genes in PPA treatment at 5 h, 10 h and 24 h by screen conditions (Gene Ontology term): Defense, SA, and ROS. **B**, Expression levels of indicated genes in response to 40 μM PPA treatment for 2 days in 20-day-old *Arabidopsis* leaves. Total RNA was extracted for qRT-PCR. *ACT2* (At3g18780) was used as an internal control. Gene expression values are presented relative to average levels in distilled-water (DW) treated leaves (set as 1). The statistical significance of the difference was confirmed by Student's *t*-test (*P<0.05, **P<0.01). Data represent the means ±SE from triplicate reactions in each experiment. This experiment was repeated three times with similar results using independent samples. The primers used for this analysis are provided in S3 Table.

We further performed a microarray experiment to identify gene expression changes induced by PPA. We harvested wild-type *Arabidopsis* leaf tissues at 0, 5, 10 and 24 h after PPA treatment. PPA-responsive genes were identified based on both significance (ANOVA p-value <0.05) and a change in expression of more than 2-fold (Log_2_>1). Compared with 0 h, we detected dramatic gene expression alterations along the time course, especially for genes related to defenses, ROS and SA (Fig 2A). The array data indicated that as early as 5 h after PPA treatments, more than 400 defense/ROS/SA related genes were up-regulated; the number of up-regulated genes increased to 616 at 24 h (Fig 2A). Gene clusters (by Gene Ontology terms) are shown in S2 Table. Table 1 shows genes highly-expressed at 24 h after PPA treatment, annotated from the TAIR database (http://www.arabidopsis.org/). Interestingly, some PPA-responsive genes were positively associated with auxin transport and root development (Table 1), indicating potential effects of PPA on root phenotypes.

**Table 1:** The cluster of selected high expression genes in microarray data at 24 h after PPA treatment.

TAIR ID	ANNOTATION	Log2 ratio
**Defense and SA**		
AT3G01830 AT3G01830	putative calcium-binding protein CML40	9.22
AT4G04490 CRK36	cysteine-rich receptor-like protein kinase 36	8.98
AT1G19250 FMO1	flavin-dependent monooxygenase 1	9.22
AT1G78410 AT1G78410	VQ motif-containing protein	8.05
AT4G02380 SAG21	senescence-associated protein	5.99
AT3G13100 ABCC7	ABC transporter C family member 7	7.4
AT3G26830 PAD3	Bifunctional dihydrocamalexate synthase/camalexin synthase	5.62
AT1G72900 AT1G72900	Toll-Interleukin-Resistance domain-containing protein	4
AT3G50930 BCS1	cytochrome BC1 synthesis	5.68
AT2G13810 ALD1	AGD2-like defense response protein 1	6.74
AT4G11890 ARCK1	protein kinase family protein	5.17
AT1G74360 AT1G74360	putative LRR receptor-like serine/threonine-protein kinase	4.09
AT3G28510 AT3G28510	AAA-type ATPase family protein	11.28
AT4G23150 CRK7	cysteine-rich receptor-like protein kinase 7	7.05
AT4G10500 AT4G10500	oxidoreductase, 2OG-Fe(II) oxygenase family protein	9.03
AT1G80840 WRKY40	putative WRKY transcription factor 40	5.03
AT5G22570 WRKY38	putative WRKY transcription factor 38	5.36
AT4G23810 WRKY53	putative WRKY transcription factor 53	4.56
AT2G46400 WRKY46	putative WRKY transcription factor 46	4.91
AT4G39030 EDS5	enhanced disease susceptibility 5	4.13
AT1G30900 VSR6	vacuolar sorting receptor 6	5.03
AT3G48090 EDS1	enhanced disease susceptibility 1 protein	3.58
AT1G74710 EDS16	Isochorismate synthase 1	3.64
AT1G13470 AT1G13470	hypothetical protein	9.03
AT3G52430 PAD4	protein PHYTOALEXIN DEFICIENT 4	3.71
AT1G64280 NPR1	Regulatory protein NPR1	1.34
AT5G45110 NPR3	NPR1-like protein 3	2.53
AT5G13320 PBS3	4-substituted benzoates-glutamate ligase GH3.12	9.18
AT3G11340 UGT76B1	UDP-dependent glycosyltransferase 76B1	9.81
AT5G57220 CYP81F2	cytochrome P450, family 81, subfamily F, polypeptide 2	4.58
AT1G35230 AGP5	arabinogalactan protein 5	10.39
AT5G42380 CML37	calcium-binding protein CML37	8.51
AT5G41740 AT5G41740	TIR-NBS-LRR class disease resistance protein	7.09
AT3G23250 MYB15	myb domain protein 15	7.03
AT1G68620 AT1G68620	probable carboxylesterase 6	8.67
AT4G23190 CRK11	cysteine-rich receptor-like protein kinase 11	5.03
AT4G23210 CRK13	cysteine-rich receptor-like protein kinase 13	4.59
AT1G34180 NAC016	NAC domain containing protein 16	6.1
AT1G72920 AT1G72920	Toll-Interleukin-Resistance domain-containing protein	2.37
AT1G75040 PR5	pathogenesis-related protein 5	5.08
AT1G33950 AT1G33950	avirulence induced protein	5.61
AT3G23110 RLP37	receptor like protein 38///receptor like protein 37	5.37
AT3G63380 AT3G63380	putative calcium-transporting ATPase 12	6.02
AT1G35710 AT1G35710	putative leucine-rich repeat receptor-like protein kinase	4.58
AT2G14610 PR1	pathogenesis-related protein 1	7.22
AT3G01080 WRKY58	WRKY DNA-binding protein 58	4.34
AT2G30770 CYP71A13	cytochrome P450, family 71, subfamily A, polypeptide 13	4.97
**ROS**		
AT1G26420 AT1G26420	FAD-binding and BBE domain-containing protein	8.49
AT5G24110 WRKY30	WRKY DNA-binding protein 30	7.83
AT1G28480 GRX480	glutaredoxin-GRX480	7.19
AT3G09940 MDHAR	monodehydroascorbate reductase (NADH)	7.28
AT1G02930 GSTF6	Glutathione S-transferase 6///glutathione S-transferase 7/11	4.19
AT5G64120 PRX71	peroxidase 71	2.96
AT1G21520 AT1G21520	hypothetical protein	6.46
AT5G47910 RBOHD	respiratory burst oxidase-D	2.05
AT1G64060 RBOHF	respiratory burst oxidase-F	1.73
AT3G49120 PRXCB	peroxidase 34///peroxidase 33	2.02
AT1G74310 HSP101	heat shock protein 101	4.1
AT2G37430 ZAT11	zinc finger protein ZAT11	5.91
AT4G26120 AT4G26120	regulatory protein NPR2	4.97
AT1G14870 PCR2	cadmium resistance protein 1///cadmium resistance protein 2	6.27
AT4G20830 AT4G20830	FAD-binding Berberine family protein	3.79
**Auxin and root development**		
AT2G47000 ABCB4	auxin efflux transmembrane transporter MDR4	1.85
AT2G17500 AT2G17500	auxin efflux carrier family protein	2.74
AT3G12830 SAUR72	SAUR-like auxin-responsive protein	4.86
AT1G56150 SAUR71	SAUR-like auxin-responsive protein	3.11
AT3G02260 BIG	auxin transport protein BIG	0.99
AT5G35735 AT5G35735	putative auxin-responsive protein	1.04
AT1G30850 RSH4	protein root hair specific 4	5.53
AT3G13870 RHD3	Root hair defective 3	1.17

To confirm these changes, we also selected several up-regulated genes and measured their transcript levels by real-time quantitative RT-PCR (Fig 2B). We examined expression of *PR* genes, and *PAD3* and *CYP71A13*, which are key genes in camalexin synthesis, and other defense-related genes such as *WRKY33*, *WRKY40*, *FMO1*, *AGP5*, *PBS3* and *ALD1*. We also measured SA-related genes, such as *PAD4*, *EDS1* and *SID2*, and ROS related genes, such as *GST1*, *APX1*, *Prx33*, *Prx34*, *RbohD* and *RbohF*. These genes were significantly up-regulated after PPA treatments (Fig 2B), supporting the microarray data. We also compared the gene expression levels in BTH- and PPA-treated leaves and found that several genes, such as *PR1*, *FMO1* and *ALD1*, showed higher expression in PPA-treated leaves (S2A Fig). Taken together, our results clearly showed that pretreatment with PPA triggered robust expression of defense-related genes.

### PPA stimulates plant resistance to bacterial infection

Antimicrobial activity screening *in vitro* revealed that the PPA compound and the traditional plant activator BTH have no direct antimicrobial activity against *Pseudomonas syringae* (S1F Fig) and *Botrytis cinerea* spore germination (S1G Fig).

**Fig 3:**
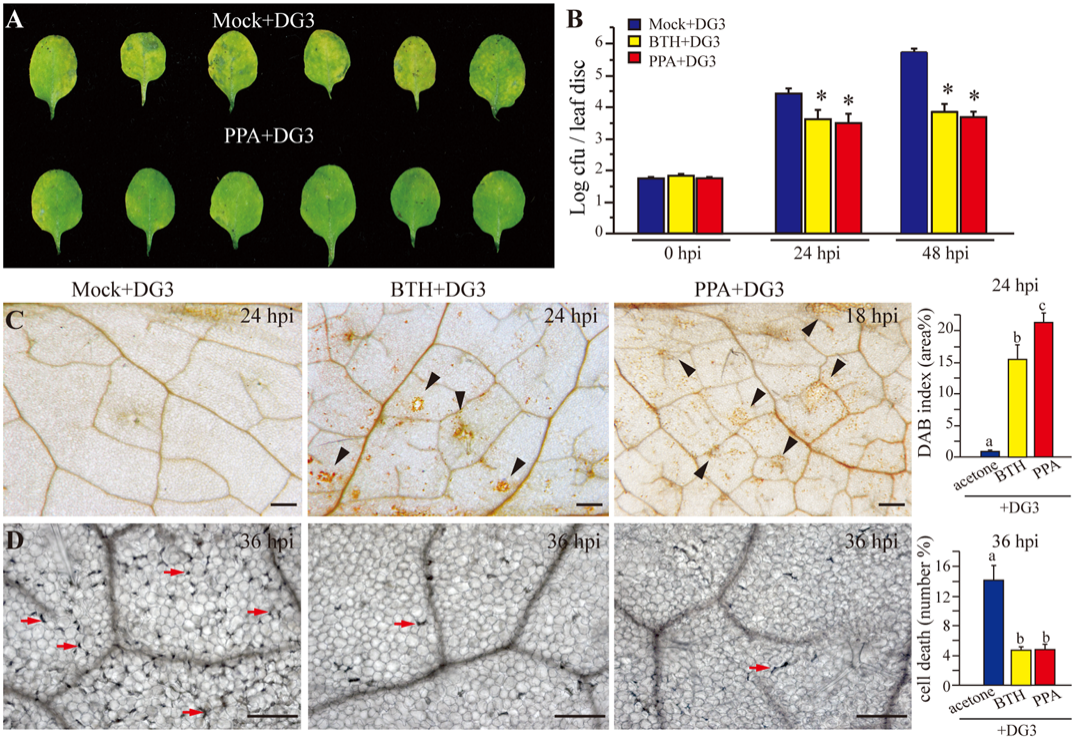
PPA triggered resistance responses after bacterial infection. **A**, Disease symptoms of 19-day-old leaves pretreated with 40 μM PPA for 2 days, followed by infiltration of 10 mM MgSO_4_ (Mock) or *Pseudomonas syringae* pv. *maculicola* strain DG3 (DG3) (OD_600_ = 0.001) 72 h post inoculation. **B**, Leaf discs were harvested at 0 dpi, 1 dpi and 2 dpi and monitored for bacterial growth. Asterisks indicate P<0.05 using Fisher's PLSD. This experiment was repeated at least six times with similar results. **C** and **D**, Microscopy images of DAB staining (**C**) and trypan blue staining (**D**). Twenty-two-day-old leaves pretreated with 0.3% acetone (mock), 100 μM BTH for 3 days and 40 μM PPA for 2 days, then infiltrated with *P*. *syringae* DG3 (OD_600_ = 0.005) for the indicated times. Note yellow DAB deposits (black arrowheads) and dead cells (red arrows). Bar = 200 μm. The right panel in (**C**) shows quantification of DAB deposit area measured by ImageJ. The right panel in (**D**) shows numbers of dead cells calculated by manual count in the field of view. Error bars represent the means ±SE from six replicates in each experiment, and data sets marked with different letters indicate significant differences (P<0.05, PLSD-test). This experiment was repeated at least three times.

**Fig 4:**
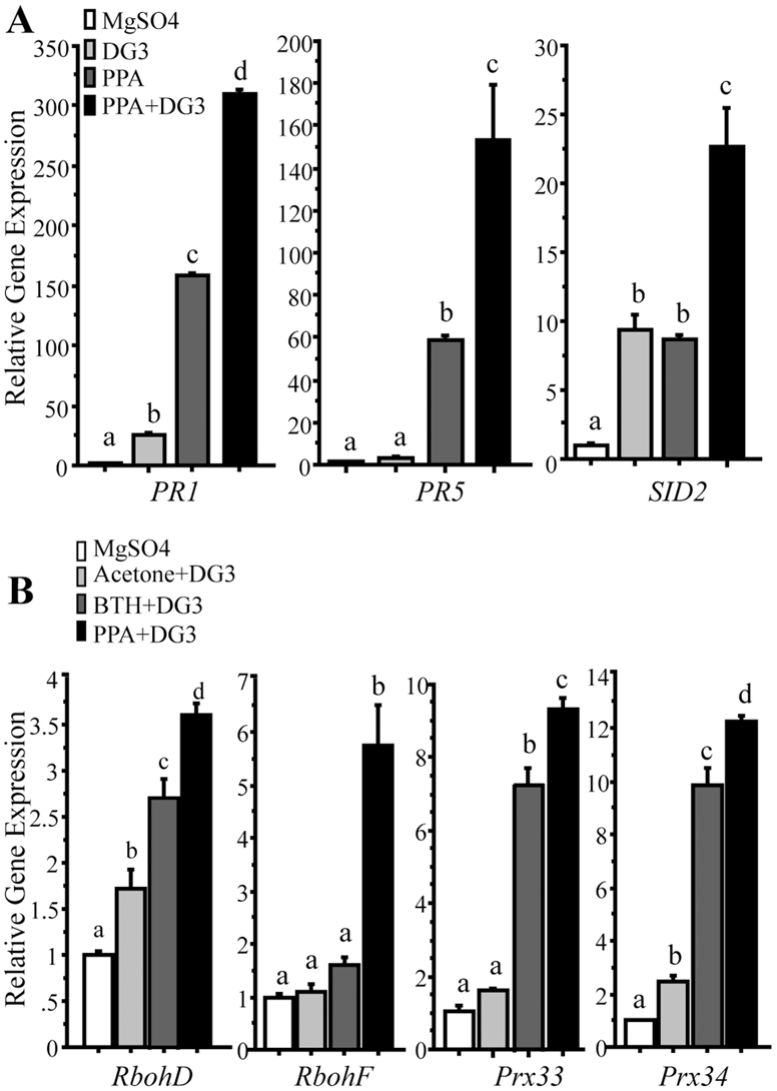
Defense and ROS related gene expression in PPA-pretreated leaves infected with bacteria. Nineteen-day-old leaves pretreated with 40 μM PPA (**A** and **B**) for 2 days and 100 μM BTH (**B**) for 3 days, then infiltrated with 10 mM MgSO_4_, 0.3% acetone (solvent of BTH) and DG3 (OD_600_ = 0.005) for 12 h. Total RNA was extracted for qRT-PCR. *ACT2* (At3g18780) was used as an internal control. Gene expression values are presented relative to average MgSO_4_ treated leaf levels (set as 1). Error bars represent the means ±SE from triplicate reactions in each experiment. Data sets marked with different letters indicate significant differences (P<0.05, PLSD-test). This experiment was repeated three times with similar results. The primers used for this analysis are provided in S3 Table.

To confirm plant resistance induced by PPA, we applied a moderate concentration of BTH (100 μM) and PPA (40 μM), and then inoculated the treated plants with pathogen. With a low dose of *P*. *syringae*, at 72 h post inoculation, PPA-pretreated leaves demonstrated reduced disease symptoms compared to mock-treated leaves (Fig 3A). Bacterial growth was reduced significantly in both BTH and PPA pretreated leaves (Fig 3B). ROS accumulation detected by DAB staining occurred in a larger area of PPA-pretreated leaves at 18 hpi (Fig 3C). At 24 hpi, PPA-pretreated leaves showed significantly increased DAB deposits compared with BTH-pretreated leaves (Fig 3C, the right panel), demonstrating a stronger, and earlier ROS burst in PPA-pretreated plants subsequently infected with bacteria. Trypan blue staining indicated that the BTH and PPA-pretreated leaves had fewer dead cells (Fig 3D), indicating that PPA and BTH can effectively protect plant cells against bacterial infection. The defense-related genes *PR1* and *PR5*, and the SA synthesis gene *SID2* showed significantly higher expression at 12 hpi in PPA-treated samples (Fig 4A). Compared with BTH pretreatment, most of the detected defense-related genes showed similar or higher expression in PPA-treated samples (S2 Fig). Strikingly, we found that at the early infection stage, transcript levels of *RbohD* and *RbohF*, as well as *Prx33* and *Prx34* were significantly higher in PPA-treated leaves than in BTH-pretreated leaves (Fig 4B), coincident with the increase in DAB staining. *PR* gene expression also increased both in PPA and BTH-pretreated leaves (S2B Fig).

Taken together, these results proved that PPA can induce plant defense responses to bacterial infection, but has no direct effect on bacteria; instead, PPA produces a higher level ROS burst, less cell death and high-level *PR* gene expression in infected leaves.

### PPA induces a strong and early ROS burst in response to bacterial infection

**Fig 5:**
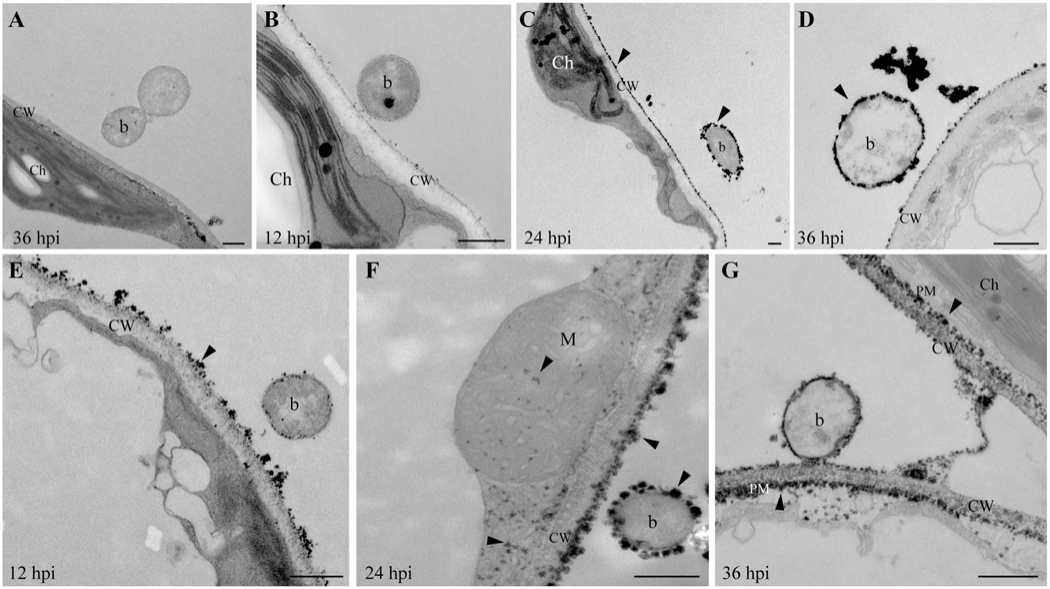
H_2_O_2_ in PPA-pretreated leaves infected with bacteria. Twenty-day-old leaves pretreated with 0.3% acetone (Mock, the solvent for BTH), 100 μM BTH for 3 days, or 40 μM PPA for 2 days, then infiltrated with *P*. *syringae* (DG3, OD_600_ = 0.005) for the indicated times. The infiltrated leaves were collected and incubated in CeCl_3_ as described in the Methods. **A**, Cell morphology of mock-treated leaves at 36 h post bacterial inoculation. Note the cerium-free bacteria. **B**–**D,** TEM images of BTH-treated leaves after DG3 inoculation for 12 h (**B**), 24 h (**C**), 36 h (**D**). **E**–**G**, TEM images of PPA-treated leaves after DG3 inoculation for 12 h (**E**), 24 h (**F**), 36 h (**G**). Arrowheads indicate cerium deposits. Ch, chloroplast; CW, Cell wall; M, Mitochondrion; PM, Plasma membrane; b, bacterium. Bar = 500 nm. This experiment was repeated at least two times and at least 6 different leaves were used in each time course.

To investigate the precise timing and localization of ROS in PPA-pretreated plants infected with bacteria, we performed cerium chloride staining to observe ROS production at the ultrastructural level. No cerium deposits were found in MgSO_4_-treated control and PPA-treated, uninfected plants (S3A Fig). Few H_2_O_2_ signals were observed in *P*. *syringae* inoculated plants at 36 hpi (Fig 5A). As early as 12 hpi, clear H_2_O_2_ signals were found in PPA-pretreated samples, indicating an earlier and stronger response than in BTH-pretreated samples (Fig 5). Both PPA- and BTH-pretreated samples showed heavy H_2_O_2_ accumulation on the plant cell wall and the surface of bacteria at 24 hpi (Fig 5C and 5F). At 36 hpi, some bacteria lost their electron-dense character and showed black round loops in intercellular spaces, indicating that these bacteria were dead (Fig 5D and 5G). Interestingly, in addition to cell wall cerium deposits, PPA-pretreated samples also showed cerium accumulation at the plasma membrane, mitochondria and cytosol (Fig 5F and 5G and S3B Fig), which differed from BTH pretreatment. The results indicated that PPA might trigger different sources of ROS in response to bacterial attack when compared with BTH. Combined with our findings on expression of ROS-related genes such as *RbohD*, *RbohF*, and *Prx33*, *Prx34* (Fig 4B), we speculate that ROS production maybe play an important role in PPA-induced defense responses, which protect plants from pathogen infection.

## Discussion

In this report, we identified a plant activator, called PPA, which induced *Arabidopsis* resistance responses against pathogen attack. An investigation of the relationship between structure and activity showed that the carboxylate group on the phenyl ring is critical to the biological activity of BTH, and the higher the molecular weight of the carboxylic acid derivative, the lower the activity of the compound in inducing defenses [34]. PPA belongs to the pyridyl-pyrimid derivative family and thus is remarkably different from other, known plant activators. Animal studies showed that pyridyl-pyrimid derivatives could inhibit glycogen synthase kinase-3β protein, indicating the importance of further investigation of pyridyl-pyrimid derivatives in the search for novel drugs [35]. Connection between BTH chemical structure and ability to induce defenses is relatively clear, but the effect of pyridyl-pyrimid like compounds on plant resistant system is still blank.

BTH treatment causes a reduction of growth [16]. By contrast, we found that PPA treatment, at a low dose, has no negative effect on *Arabidopsis* and rice plant vegetative growth and, in fact, promote a slight increase in size and fresh weight. This effect may provide an advantage in agricultural crops, if PPA can induce plant defenses against pathogenic invasion and also maintain production. In addition, compared with BTH-treated seedlings, PPA treatment caused less of an effect on the elongation of main roots. Also, the PPA-treated plants had significantly more lateral roots, an effect not be reported for other plant activators. The plant lateral root formation is profoundly affected by auxin transport, that is in favor of water and nutrient transport into plant tissues [36]. On the other hand, the phytotoxicity of a relative high concentration of PPA (such as 80 μM) reminds us that the working PPA concentration could vary for different plant species and periods of development.

The microarray data and qRT-PCR analysis showed that large numbers of ROS-related genes were activated as early as 5 h after PPA treatments and increases two-fold at 10 h. We observed highly significant activation of *FMO1* (*flavin-dependent monooxygenase*). A comparison with published BTH microarray data [37] revealed that the expression pattern induced by BTH partially differs from the pattern induced by PPA, with no significant changes in the expression of *FMO1*. *FMO1* may have a pivotal role SA synthesis in systemic tissue [38] and the SA-independent, EDS1-regulated defense pathway [39]. The *fmo1* mutant is defective only in SA accumulation in systemic tissue and in SAR, and has normal local SA synthesis and ETI [38]. We speculate that PPA may induce SAR in *Arabidopsis*, based on the program of gene expression we observed. In addition, we also found several auxin and root development genes expressed after PPA treatments, which may explain why PPA did not inhibit plant growth and root system development.

ROS is an early, significant signal in the *Arabidopsis* defense system, and activation of resistance responses in plants is associated with a parallel burst of ROS [40]. Our data revealed that PPA-pretreated plants showed earlier and more DAB deposition and cerium deposits than BTH pre-treated plants, following bacterial infection. Interestingly, the cerium encircled the bacteria, which eventually died, indicating that a high-level ROS burst may directly kill the invading bacteria. The mechanisms by which PPA induces high-level ROS accumulation remain to be investigated.

Traditional pesticides directly kill pathogens to protect plants. To address whether PPA has a direct effect on microbes, we checked if it affected the reproduction of bacteria or the germination of fungal spores. Our results showed that the PPA working concentration had no visible direct effect on pathogen growth or germination. This is the main difference between plant activators and traditional pesticides. PPA combines significant induction of plant defense responses and promotion of plant biomass; therefore, we conclude that PPA is a novel plant activator and may suitable for applications in crop production.

## Supporting Information

S1 Fig Comparison of PPA with other plant activators for chemical structure and effect on plant growth. **A**, Chemical structures of PPA, BTH, INA, PBZ and BABA. **B** and **C**, A concentration of 40 μM PPA was suitable for plants. Eighteen-day-old plants were sprayed with 40 μM PPA, 300 μM BTH or 0.3% acetone (Control) for 9 days. Trypan blue staining was used for cell death detection (**B**). DAB staining was used for ROS detection (**C**). **D**, Photos of seedlings grown on 1/2x MS plates containing 0.3% acetone as a control, 40 μM PPA or 300 μM BTH for 20 days. **E**, Height comparison of rice seedlings after BTH and PPA treatments. Three-day-old germinated rice seedlings were put on 0.15% agar containing 0.1% acetone (control), 100 μM BTH or 4 μM PPA under greenhouse conditions and photographed 14 days later. Data sets marked with different letters indicate significant differences (P<0.05, PLSD-test). Error bars represent the means ±SE (n = 30). This experiment was repeated twice with similar results. **F** and **G,** The impact of PPA on bacterial and fungal growth. Bacteria were cultured in King's B liquid medium and treated with 40 μM PPA or 300 μM BTH for the indicated times. The OD600 was measured every 2 h (**F**). For *Botrytis cinerea* spore germination (**G**), 2×10^7^ spores (10 μL) were germinated on glass slides covered with 1% agar containing 40 μM PPA or 300 μM BTH. The spore germination was calculated at 12 h after treatments. Error bars represent the means ±SE from three repeat experiments.
(TIF)

S2 Fig Gene expressions in PPA or BTH treated plants. **A**, Expression levels indicate changes in transcript levels in response to treatment with 40 μM PPA (2 days) and 300 μM BTH (3 days) in 21-day-old *Arabidopsis* leaves. Total RNA was extracted for qRT-PCR. *ACT2* (At3g18780) was used as an internal control. Gene expression values are presented relative to average distilled-water (DW) treated leaf levels (set as 1). Acetone (0.3%) is the solvent for BTH. **B**, Measurement of resistance gene expression levels after bacterial infection used the same samples as shown in Fig 4B. Gene expression values are presented relative to average levels in MgSO_4_-treated leaves (set as 1). Data sets marked with different letters indicate significant differences (P<0.05, PLSD-test). This experiment was repeated three times with similar results. The primers used for this analysis are provided in S3 Table.
(TIF)

S3 Fig H_2_O_2_ localization in PPA-pretreated leaves using the cerium chloride method. **A**, Cell morphology of 40 μM PPA-treated 19-day-old leaves for 96 h. No cerium deposits were found in cells. **B**, PPA-pretreated leaves were injected with *P*. *syringae* DG3 (OD600 = 0.005) for 24 h. Cerium deposits (arrowheads) were observed on the cell wall (CW) and in mitochondria (M). Bar = 500 nm.
(TIF)

S1 Table The cluster of selected high expression genes in microarray data after PPA treatments. (PDF)

S2 Table Screen conditions of GO term. (PDF)

S3 Table Primers used in this study. (PDF)
