# Supplementary figures and images for: A Novel Pyrimidin-Like Plant Activator Stimulates Plant Disease Resistance and Promotes Growth
Source: PLoS One. 2015 Apr 7;10(4):e0123227. doi: 10.1371/journal.pone.0123227 (PMC4388471; doi:10.1371/journal.pone.0123227)

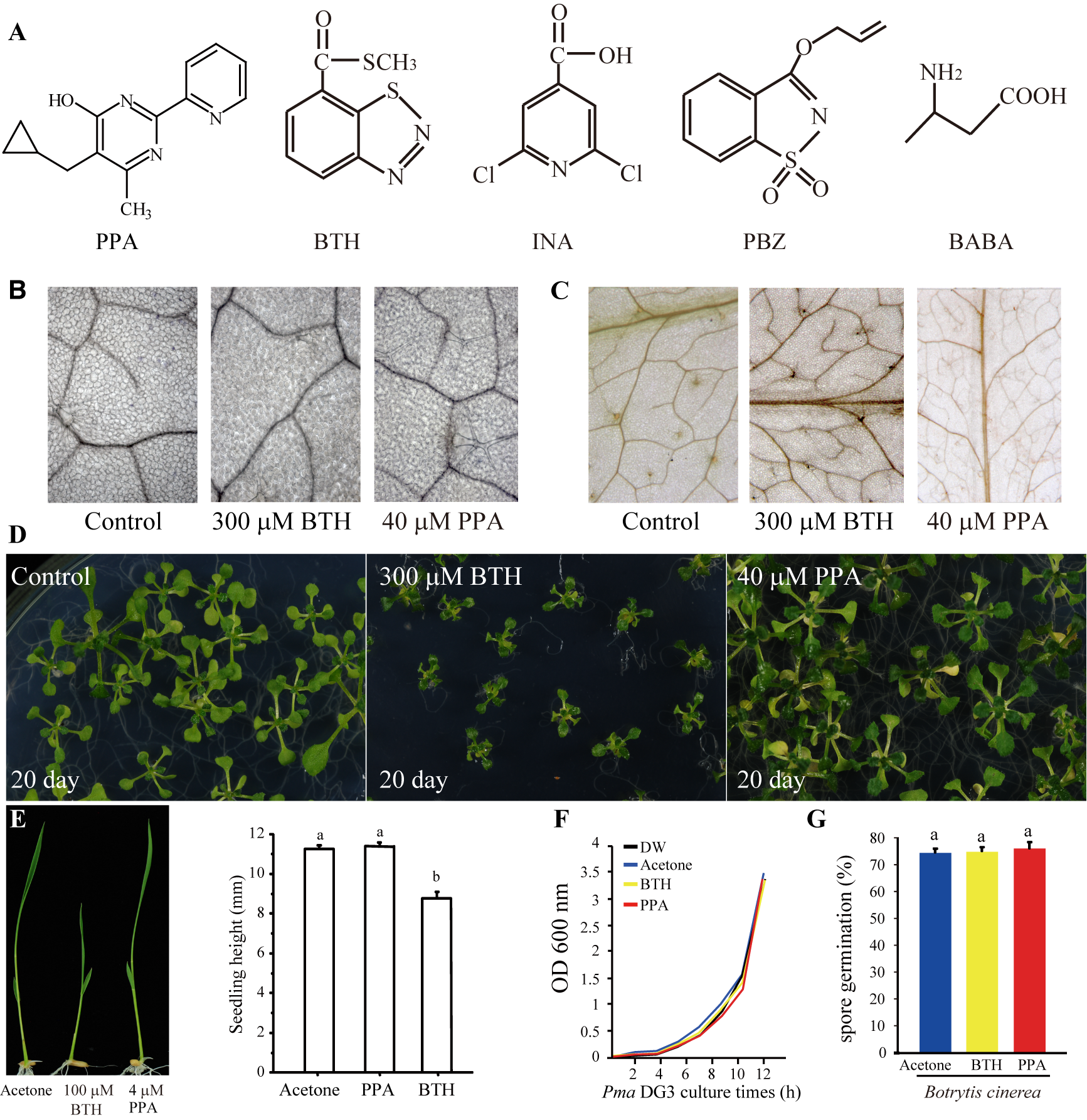

Supplement: S1 Fig — A, Chemical structures of PPA, BTH, INA, PBZ and BABA. B and C, A concentration of 40 μM PPA was suitable for plants. Eighteen-day-old plants were sprayed with 40 μM PPA, 300 μM BTH or 0.3% acetone (Control) for 9 days. Trypan blue staining was used for cell death detection (B). DAB staining was used for ROS detection (C). D, Photos of seedlings grown on 1/2x MS plates containing 0.3% acetone as a control, 40 μM PPA or 300 μM BTH for 20 days. E, Height comparison of rice seedlings after BTH and PPA treatments. Three-day-old germinated rice seedlings were put on 0.15% agar containing 0.1% acetone (control), 100 μM BTH or 4 μM PPA under greenhouse conditions and photographed 14 days later. Data sets marked with different letters indicate significant differences (P<0.05, PLSD-test). Error bars represent the means ±SE (n = 30). This experiment was repeated twice with similar results. F and G, The impact of PPA on bacterial and fungal growth. Bacteria were cultured in King's B liquid medium and treated with 40 μM PPA or 300 μM BTH for the indicated times. The OD600 was measured every 2 h (F). For Botrytis cinerea spore germination (G), 2×10^7^ spores (10 μL) were germinated on glass slides covered with 1% agar containing 40 μM PPA or 300 μM BTH. The spore germination was calculated at 12 h after treatments. Error bars represent the means ±SE from three repeat experiments. (TIF) [file pone.0123227.s001.tif]

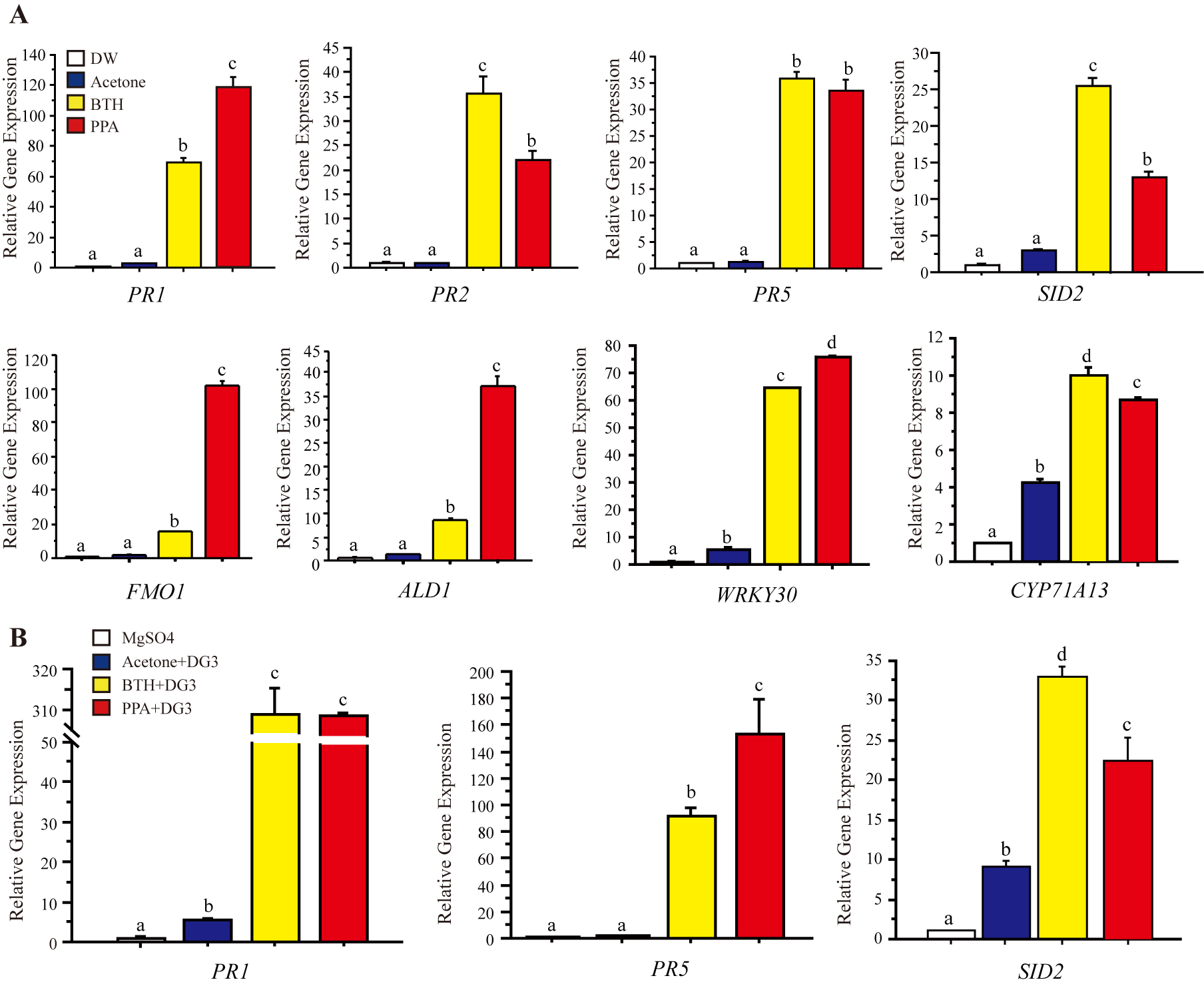

Supplement: S2 Fig — A, Expression levels indicate changes in transcript levels in response to treatment with 40 μM PPA (2 days) and 300 μM BTH (3 days) in 21-day-old Arabidopsis leaves. Total RNA was extracted for qRT-PCR. ACT2 (At3g18780) was used as an internal control. Gene expression values are presented relative to average distilled-water (DW) treated leaf levels (set as 1). Acetone (0.3%) is the solvent for BTH. B, Measurement of resistance gene expression levels after bacterial infection used the same samples as shown in Fig 4B. Gene expression values are presented relative to average levels in MgSO4-treated leaves (set as 1). Data sets marked with different letters indicate significant differences (P<0.05, PLSD-test). This experiment was repeated three times with similar results. The primers used for this analysis are provided in S3 Table. (TIF) [file pone.0123227.s002.tif]

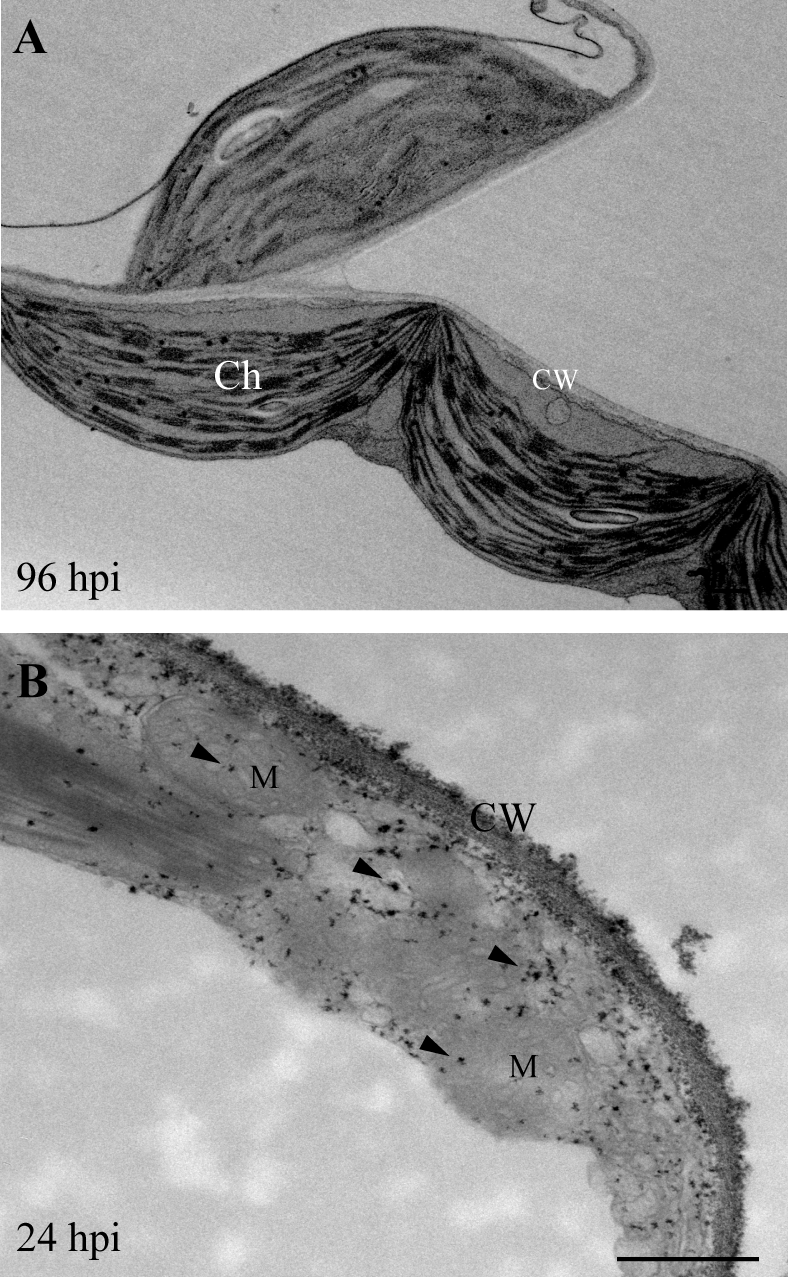

Supplement: S3 Fig — A, Cell morphology of 40 μM PPA-treated 19-day-old leaves for 96 h. No cerium deposits were found in cells. B, PPA-pretreated leaves were injected with P. syringae DG3 (OD600 = 0.005) for 24 h. Cerium deposits (arrowheads) were observed on the cell wall (CW) and in mitochondria (M). Bar = 500 nm. (TIF) [file pone.0123227.s003.tif]
