# Supplementary material for: A Novel Pyrimidin-Like Plant Activator Stimulates Plant Disease Resistance and Promotes Growth
Source: PLoS One. 2015 Apr 7;10(4):e0123227. doi: 10.1371/journal.pone.0123227 (PMC4388471; doi:10.1371/journal.pone.0123227)
Supplement: S1 Table — (PDF) [file pone.0123227.s004.pdf]

annotation			function			expression		
TAIRid	symbol	description	defense	SA	ROS	PPA5h	PPA10h	PPA24h
AT2G37430	ZAT11	zinc finger protein ZAT11	+	-	+	8.15	8.22	5.91
AT1G51800	IOS1	putative leucine-rich repeat protein kinase	+	-	+	7.92	8.27	7.59
AT3G11340	UGT76B1	UDP-dependent glycosyltransferase 76B1	+	-	-	7.77	11.41	9.81
AT3G01830	AT3G01830	putative calcium-binding protein CML40	+	+	+	7.44	9.24	9.22
AT4G04490	CRK36	cysteine-rich receptor-like protein kinase 36	+	+	-	7.26	9.51	8.98
AT5G57220	CYP81F2	cytochrome P450, family 81, subfamily F, polypeptide 2	+	-	-	7.11	6.51	4.58
AT5G24110	WRKY30	WRKY DNA-binding protein 30	-	+	+	6.67	6.94	7.83
AT1G26420	AT1G26420	FAD-binding and BBE domain-containing protein	+	-	+	6.59	8.86	8.49
AT5G42380	CML37	calcium-binding protein CML37	+	-	-	6.38	7.69	8.51
AT4G21390	B120	G-type lectin S-receptor-like serine/threonine-protein kinase B120	+	+	+	6.37	6.53	4.8
AT1G05680	UGT74E2	Uridine diphosphate glycosyltransferase 74E2	-	-	+	6.22	6.35	3.4
AT4G39670	AT4G39670	glycolipid transfer protein	+	+	+	6.13	7.53	7.33
AT1G13470	AT1G13470	hypothetical protein	+	+	+	6.07	8.6	9.03
AT1G78410	AT1G78410	VQ motif-containing protein	+	+	+	6.01	7.85	8.05
AT5G51440	AT5G51440	heat shock protein 23.5	-	-	+	5.99	6.58	2.48
AT1G19250	FMO1	flavin-dependent monooxygenase 1	+	-	-	5.71	10.41	9.22
AT5G41740	AT5G41740	TIR-NBS-LRR class disease resistance protein///TIR-NBS-LRR class disease resistance protein	+	-	-	5.63	7.05	7.09
AT3G63380	AT3G63380	putative calcium-transporting ATPase 12	+	-	+	5.51	7.48	6.02
AT5G13320	PBS3	4-substituted benzoates-glutamate ligase GH3.12	+	+	+	5.32	8.89	9.18
AT4G02380	SAG21	senescence-associated protein	+	+	+	5.3	6.4	5.99
AT1G28480	GRX480	glutaredoxin-GRX480	+	+	+	5.21	7.14	7.19
AT3G23250	MYB15	myb domain protein 15	+	-	+	5.21	6.37	7.03
AT3G11080	RLP35	receptor like protein 35	+	-	-	A	A	5.58
AT3G13100	ABCC7	ABC transporter C family member 7	-	+	-	5.17	7.67	7.4
AT2G26150	HSFA2	heat stress transcription factor A-2	-	-	+	5.16	5.4	5.87
AT4G16260	AT4G16260	putative beta-1,3-endoglucanase that interacts with the 30C02 cyst nematode effector. May pla	+	-	-	5.03	6.02	4.46
AT1G71000	AT1G71000	chaperone DnaJ-domain containing protein	-	-	+	A	7.19	A
AT1G80840	WRKY40	putative WRKY transcription factor 40	+	+	+	5.01	5.28	5.03
AT3G09940	MDHAR	monodehydroascorbate reductase (NADH)	-	-	+	4.82	7.21	7.28
AT4G23190	CRK11	cysteine-rich receptor-like protein kinase 11	+	-	+	4.77	4.97	5.03
AT3G28740	CYP81D11	cytochrome P450 CYP81D11	+	-	-	4.76	3.87	1.89
AT4G35180	LHT7	LYS/HIS transporter 7	+	-	-	4.73	6.86	6.3
AT5G22530	AT5G22530	hypothetical protein	-	+	-	4.69	7.48	5.66
AT1G19020	AT1G19020	hypothetical protein	+	+	+	4.67	5.67	5.27
AT2G44960	HUB1	E3 ubiquitin-protein ligase BRE1-like 1	+	-	+	A	A	4.71
AT1G27730	STZ	zinc finger protein STZ/ZAT10	+	-	+	4.61	4.95	5.07
AT2G03760	SOT12	sulphotransferase 12	+	+	+	4.52	4.38	1.38
AT5G13330	Rap2.6L	ethylene-responsive transcription factor ERF113	-	+	-	A	A	6.99
AT2G35980	YLS9	late embryogenesis abundant hydroxyproline-rich glycoprotein	+	+	+	4.49	6.76	7.79
AT3G26830	PAD3	bifunctional dihydrocamalexate synthase/camalexin synthase	+	+	+	4.46	6.99	5.62
AT1G72900	AT1G72900	Toll-Interleukin-Resistance domain-containing protein	+	+	-	4.39	4.96	4
AT4G25200	HSP23.6-MI	small heat shock protein 23.6	-	-	+	4.38	4.44	A
AT3G25250	AGC2-1	AGC (cAMP-dependent, cGMP-dependent and protein kinase C) kinase family protein	-	-	+	4.34	5.05	3.75
AT2G32140	AT2G32140	transmembrane receptor protein	+	-	-	4.33	6.51	7.02

AT2G18690	AT2G18690	hypothetical protein	+	+	-	4.29	6.25	5.95
AT3G50930	BCS1	cytochrome BC1 synthesis	+	+	-	4.27	5.72	5.68
AT1G65390	PP2-A5	protein PHLOEM protein 2-LIKE A5	+	-	-	4.26	A	A
AT4G17490	ERF6	ethylene responsive element binding factor 6	+	-	+	4.24	3.79	2.25
AT1G63490	AT1G63490	transcription factor jumonji (jmjC) domain-containing protein	-	-	+	4.16	4.07	A
AT1G16420	MC8	metacaspase 8	-	-	+	4.13	5.98	6.18
AT3G57240	BG3	beta-1,3-glucanase 3	+	+	+	4.09	5.12	3.14
AT5G52760	AT5G52760	copper transport family protein	+	+	+	4.07	5.69	6.13
AT4G14365	XBAT34	putative E3 ubiquitin-protein ligase XBAT34	+	+	+	4.05	5.95	5.68
AT1G21250	WAK1	wall-associated receptor kinase 1	+	+	-	4	5.15	5.02
AT3G57460	AT3G57460	catalytic/ metal ion binding / metalloendopeptidase/ zinc ion binding protein	-	+	-	3.98	5.07	4.97
AT2G32030	AT2G32030	GCN5-related N-acetyltransferase-like protein	+	-	+	3.95	4.21	3.99
AT2G24850	TAT3	tyrosine aminotransferase 3	-	+	-	3.95	6.86	6.69
AT2G13810	ALD1	AGD2-like defense response protein 1	+	+	-	A	7.3	6.74
AT3G47460	ATSMC2	structural maintenance of chromosomes protein 2-2	+	-	-	A	3.73	A
AT2G26560	PLA2A	phospholipase A 2A	+	+	+	3.74	4.71	1.75
AT4G11170	RMG1	putative disease resistance protein	+	-	-	A	5	4.98
AT2G29500	AT2G29500	HSP20 family protein	-	-	+	3.71	4	1.16
AT5G27420	CNI1	E3 ubiquitin-protein ligase ATL31	+	-	+	3.71	4.17	3.66
AT5G12030	HSP17.6A	heat shock protein 17.6A	-	-	+	3.69	3.16	4.46
AT1G02930	GSTF6	glutathione S-transferase 6///glutathione S-transferase 7/11	+	-	+	3.63	4.72	4.19
AT1G56520	AT1G56520	TIR-NBS-LRR class disease resistance protein	+	-	-	A	4.18	3.15
AT3G26210	CYP71B23	cytochrome P450 71B23	+	-	-	3.58	4.8	5
AT3G12580	HSP70	heat shock protein 70-4	-	-	+	3.57	5.35	3.88
AT5G52750	AT5G52750	heavy metal transport/detoxification domain-containing protein	+	-	-	3.56	3.93	4.39
AT3G01290	HIR2	SPFH/Band 7/PHB domain-containing membrane-associated protein	+	-	-	3.56	4.38	4.29
AT4G11890	ARCK1	protein kinase family protein	+	+	+	3.52	5.62	5.17
AT1G05560	UGT75B1	UDP-glucosyltransferase 75B1	-	+	-	3.52	3.37	1.21
AT1G02360	AT1G02360	putative chitinase	+	-	+	3.5	4.55	4.07
AT1G74310	HSP101	heat shock protein 101	-	-	+	3.49	4.53	4.1
AT5G05300	AT5G05300	hypothetical protein	+	-	-	3.49	4.38	4.57
AT1G74360	AT1G74360	putative LRR receptor-like serine/threonine-protein kinase	+	+	+	3.48	4.22	4.09
AT4G23210	CRK13	cysteine-rich receptor-like protein kinase 13	+	-	+	3.47	5.42	4.59
AT5G20230	BCB	blue-copper-binding protein	+	-	+	3.44	4.92	5.42
AT2G43620	AT2G43620	chitinase	+	-	+	3.44	3.71	1.88
AT3G05360	RLP30	receptor like protein 30	+	+	-	A	5.16	4.6
AT5G52640	HSP90.1	heat shock protein 90.1	+	-	+	3.43	6.02	3.85
AT4G33050	EDA39	calmodulin-binding protein	+	+	+	3.39	4.47	4.38
AT2G15120		basic secretory protein family protein	+	-	-	A	3.08	A
AT3G28510	AT3G28510	AAA-type ATPase family protein	-	+	+	A	10.23	11.28
AT3G60420	AT3G60420	phosphoglycerate mutase family protein	+	+	-	3.38	5.17	5.18
AT4G15975	AT4G15975	RING-H2 finger protein ATL17	+	-	+	3.35	A	A
AT4G20860	AT4G20860	FAD-binding Berberine family protein	+	-	+	3.35	3.4	3.07
AT1G35710	AT1G35710	putative leucine-rich repeat receptor-like protein kinase	+	-	-	3.35	4.37	4.58
AT4G20830	AT4G20830	FAD-binding Berberine family protein	+	-	+	3.32	4.03	3.79
AT5G22570	WRKY38	putative WRKY transcription factor 38	+	+	+	3.28	6.14	5.36

AT5G52050	AT5G52050	MATE efflux family protein	+	-	-	3.27	3.21	3.53
AT5G59820	RHL41	high light responsive zinc finger protein ZAT12	-	-	+	3.21	4.77	4.56
AT2G47000	ABCB4	auxin efflux transmembrane transporter MDR4	-	-	+	3.12	5.11	1.85
AT1G61340	FBS1	F-box protein	-	+	-	3.09	2.71	2.31
AT1G47890	RLP7	receptor like protein 7	+	-	-	A	4.97	5.54
AT2G38470	WRKY33	putative WRKY transcription factor 33	+	+	+	3.04	3.73	3.41
AT3G48640	AT3G48640	hypothetical protein	+	+	+	3.04	5.31	5.63
AT2G29420	GSTU7	glutathione S-transferase tau 7	-	+	-	3.01	3.28	2.19
AT4G22530	AT4G22530	S-adenosyl-L-methionine-dependent methyltransferase domain-containing protein	+	-	+	3.01	3.3	1.71
AT1G72520	LOX4	lipoxygenase 4	+	-	-	3.01	4.9	4.27
AT1G66090	AT1G66090	TIR-NBS class of disease resistance protein	+	+	-	3	3.37	4.04
AT4G23220	CRK14	cysteine-rich receptor-like protein kinase 14	-	+	-	3	4.13	3.55
AT1G52200	AT1G52200	PLAC8 family protein	-	-	+	2.99	3.85	1.67
AT1G76600	AT1G76600	hypothetical protein	+	-	+	2.98	3.23	2.2
AT4G23810	WRKY53	putative WRKY transcription factor 53	+	+	+	2.96	3.88	4.56
AT1G72920	AT1G72920	Toll-Interleukin-Resistance domain-containing protein	+	-	-	2.94	2.48	2.37
AT2G16870	AT2G16870	TIR-NBS-LRR class disease resistance protein	+	-	-	A	4.59	4.7
AT4G22980	AT4G22980	hypothetical protein	+	+	-	2.9	4.17	3.33
AT1G57630	AT1G57630	Toll-Interleukin-Resistance domain-containing protein	+	+	-	2.9	5.94	5.8
AT4G23150	CRK7	cysteine-rich receptor-like protein kinase 7	-	+	-	2.9	6.84	7.05
AT3G16530	AT3G16530	legume lectin-like protein	+	-	-	2.89	2.45	1.51
AT1G09970	LRR XI-23	leucine-rich receptor-like protein kinase	+	-	+	2.89	3.29	2.45
AT5G26920	CBP60G	Cam-binding protein 60-like G	+	+	+	2.89	4.61	5.13
AT5G60950	COBL5	COBRA-like protein 5	+	+	-	2.76	3.73	4.31
AT2G14560	LURP1	LURP1 protein	+	+	+	2.74	4.45	4.4
AT1G75040	PR5	pathogenesis-related protein 5	+	+	+	2.73	4.12	5.08
AT1G14870	PCR2	cadmium resistance protein 1///cadmium resistance protein 2	-	-	+	2.71	5.67	6.27
AT4G26120	AT4G26120	regulatory protein NPR2	+	+	+	A	4.04	4.97
AT2G33710	AT2G33710	ethylene-responsive transcription factor ERF112	+	-	+	A	A	4.38
AT3G56710	SIB1	sigma factor binding protein 1	+	+	+	2.7	2.92	2.12
AT2G23680	AT2G23680	Cold acclimation protein WCOR413 family	+	+	+	2.68	3.79	3.33
AT5G25930	AT5G25930	protein kinase family protein with leucine-rich repeat domain	+	-	+	2.64	3.5	2.79
AT5G54610	ANK	ankyrin repeat family protein	+	+	+	2.63	2.78	4.13
AT5G06860	PGIP1	polygalacturonase inhibitor 1	+	+	-	2.63	2.47	2.2
AT4G08870	ARGAH2	arginase///arginine amidohydrolase	+	-	-	2.61	-0.19	1.03
AT5G54860	AT5G54860	probable folate-biopterin transporter 4	-	+	-	2.61	4.11	3.16
AT3G55980	SZF1	salt-inducible zinc finger 1	+	-	+	2.61	2.6	2.92
AT5G24780	VSP1	vegetative storage protein 1///vegetative storage protein 2	+	-	-	2.59	0.38	-1.93
AT3G23110	RLP37	receptor like protein 38///receptor like protein 37	+	-	-	2.59	4.28	5.37
AT3G13950	AT3G13950	hypothetical protein	+	+	+	2.58	4.51	5.34
AT4G14400	ACD6	protein accelerated cell death 6	+	+	-	2.58	3.32	3.24
AT1G73800		protein SAR Deficient 1	+	+	+	2.58	4.17	4.3
AT5G24210	AT5G24210	lipase class 3 family protein	-	+	-	2.57	3.98	4.56
AT1G05575	AT1G05575	hypothetical protein	+	-	+	2.57	2.81	1.94
AT5G39580	AT5G39580	peroxidase 62	+	-	-	A	3.2	3.55
AT3G13790	ATBFRUCT1	beta-fructofuranosidase, insoluble isoenzyme CWINV1	+	-	+	2.5	3.24	3.65

AT5G64120	PRX71	peroxidase 71	+	+	+	2.49	2.5	2.96
AT5G13930	TT4	chalcone synthase	-	-	+	2.48	0.47	2.87
AT1G72940	AT1G72940	Toll-Interleukin-Resistance domain-containing protein	+	-	-	2.47	1.99	2.11
AT3G12500	HCHIB	basic chitinase B	+	-	-	2.46	3.18	3.84
AT3G48080	AT3G48080	lipase class 3 family protein / disease resistance protein-related protein	-	+	-	2.45	4.29	4.59
AT5G01540	LECRKA4.1	lectin receptor kinase A4.1	+	+	-	2.45	3	4.39
AT3G14990	DJ1A	protein DJ-1-like A	-	-	+	2.43	3.35	3.67
AT1G19180	JAZ1	protein TIFY 10A	+	+	-	2.43	2.66	2.09
AT2G20560	AT2G20560	DNAJ heat shock family protein	-	-	+	2.4	2.75	3.31
AT2G39200	MLO12	MLO-like protein	+	-	+	2.4	A	1.75
AT2G26020	PDF1.2b	putative defensin-like protein	+	-	-	2.4	2.35	1.61
AT1G09080	BIP3	probable mediator of RNA polymerase II transcription subunit 37b	-	-	+	2.39	4.92	7.02
AT1G01560	MPK11	mitogen-activated protein kinase 11	+	+	+	2.38	3.93	3.67
AT1G66880	AT1G66880	serine/threonine protein kinase	+	+	+	2.38	3.76	3.53
AT4G17500	ERF-1	ethylene-responsive transcription factor 1A	+	-	+	2.37	3.28	2.91
AT1G72060	AT1G72060	serine-type endopeptidase inhibitor	-	-	+	2.36	1.99	4.26
AT4G10500	AT4G10500	oxidoreductase, 2OG-Fe(II) oxygenase family protein	-	+	-	A	4.47	9.03
AT2G29350	SAG13	senescence-associated protein 13	+	-	-	2.35	4.25	6.93
AT3G61190	BAP1	BON association protein 1	+	+	-	2.33	4.3	4.62
AT1G01060	LHY	myb-related putative transcription factor	-	+	-	2.31	-3.42	6.97
AT3G11010	RLP34	receptor like protein 53///receptor like protein 34	+	-	-	2.3	4.06	4.98
AT1G10340	AT1G10340	ankyrin repeat-containing protein	-	+	-	2.3	4.56	5.21
AT4G21400	CRK28	cysteine-rich receptor-like protein kinase 28	+	-	+	2.3	3.78	2.99
AT4G18880	HSF A4A	heat stress transcription factor A-4a	+	-	+	2.29	3.07	2.03
AT3G57700	AT3G57700	putative protein kinase	+	+	+	2.29	2.82	2.9
AT5G66890	AT5G66890	putative disease resistance protein	+	-	-	A	4.12	3.43
AT3G47480	AT3G47480	putative calcium-binding protein CML47	+	+	+	2.27	4.47	4.95
AT1G08050	AT1G08050	C3HC4-type RING finger-containing protein	+	+	-	2.27	4.56	4.44
AT3G25780	AOC3	allene oxide cyclase 3	+	+	-	2.24	3.99	3.28
AT3G52400	SYP122	syntaxin-122	+	+	+	2.22	3.24	2.96
AT1G51790	AT1G51790	leucine-rich repeat protein kinase-like protein	+	-	+	2.21	1.99	1.45
AT2G40140	CZF1	zinc finger CCCH domain-containing protein 29	+	+	+	2.19	3.59	3.18
AT4G33070	PDC1	pyruvate decarboxylase	-	+	+	2.18	6.08	A
AT2G46400	WRKY46	putative WRKY transcription factor 46	-	+	-	2.18	4.52	4.91
AT3G54420	EP3	chitinase class IV	+	-	+	2.17	3.28	3.72
AT4G39030	EDS5	enhanced disease susceptibility 5	+	+	+	2.17	4.93	4.13
AT4G23320	CRK24	cysteine-rich receptor-like protein kinase 24	+	-	-	2.17	2.97	3.65
AT5G46050	PTR3	peptide transporter 3	+	+	-	2.16	3.01	3.87
AT2G44840	ERF13	ethylene-responsive transcription factor 13	+	+	+	2.16	2.41	2.46
AT4G11280	ACS6	1-aminocyclopropane-1-carboxylate synthase 6	-	-	+	2.15	2.28	2.38
AT4G12720	NUDT7	nudix hydrolase 7	+	+	+	2.15	3.1	2.97
AT3G56400	WRKY70	WRKY transcription factor 70	+	+	+	2.15	3.76	3.74
AT1G66550	WRKY67	putative WRKY transcription factor 67	+	-	-	A	5.64	A
AT2G47130	SDR3	short-chain dehydrogenase reductase 3a	+	-	-	2.15	4.18	3.83
AT1G18570	MYB51	myb domain protein 51	+	+	+	2.14	2.63	1.02
AT1G71390	RLP11	receptor like protein 11	+	+	-	A	A	4.29

AT1G21520	AT1G21520	hypothetical protein	-	-	+	A	5.79	6.46
AT2G39210	AT2G39210	major facilitator protein	+	-	-	2.12	2.76	3.43
AT4G39890	RABH1c	Ras-related protein RABH1c	+	+	-	2.11	2.53	3.23
AT1G17745	PGDH	D-3-phosphoglycerate dehydrogenase	+	-	-	2.11	3.93	3.27
AT5G38900	AT5G38900	Thioredoxin superfamily protein	+	-	-	2.1	5.38	5.72
AT5G44420	PDF1.2	ethylene- and jasmonate-responsive plant defensin	+	+	-	2.09	2.22	0.87
AT2G30250	WRKY25	WRKY transcription factor 25	+	+	-	2.09	2.69	2.3
AT1G51660	MKK4	mitogen-activated protein kinase kinase 4	+	+	+	2.08	3.18	3.39
AT3G04720	PR4	pathogenesis-related 4	+	-	-	2.08	2.16	2.11
AT2G40750	WRKY54	WRKY DNA-binding protein 54	+	+	+	2.06	3.35	1.6
AT1G30900	VSR6	vacuolar sorting receptor 6	-	+	-	2.06	3.72	5.03
AT5G24530	DMR6	downy mildew resistance 6 protein / oxidoreductase	+	+	+	2.05	3.07	4.75
AT4G08850	AT4G08850	probable LRR receptor-like serine/threonine-protein kinase	+	-	-	2.04	2.76	3.2
AT1G52410	TSA1	TSK-associating protein 1	+	-	-	2.03	1.09	-0.03
AT1G25400	AT1G25400	hypothetical protein	+	+	-	2.03	2.02	1.7
AT2G32680	RLP23	receptor like protein 23	+	+	+	2.03	3.34	5.19
AT2G21640	AT2G21640	hypothetical protein	-	-	+	2.01	3.45	0.76
AT3G52430	PAD4	protein PHYTOALEXIN DEFICIENT 4	+	+	+	2	3.86	3.71
AT3G44400	AT3G44400	TIR-NBS-LRR class disease resistance protein	+	-	-	1.98	2.73	2.77
AT3G22160	AT3G22160	VQ motif-containing protein	-	+	-	1.98	3.39	3.59
AT5G64510	TIN1	hypothetical protein	-	-	+	1.98	3.63	3.87
AT1G52040	MBP1	myrosinase-binding protein 1///myrosinase-binding protein 2	+	-	-	1.97	-0.4	-0.28
AT4G01700	AT4G01700	Chitinase family protein	+	-	-	1.96	3.07	3.09
AT5G61600	ERF104	ethylene-responsive transcription factor ERF104	+	-	-	1.96	2.38	2.02
AT4G03450	AT4G03450	ankyrin repeat-containing protein	+	+	+	1.96	3.84	4.01
AT4G12400	Hop3	carboxylate clamp-tetratricopeptide repeat protein	-	-	+	1.94	3.07	1.47
AT1G61360	AT1G61360	G-type lectin S-receptor-like serine/threonine-protein kinase	+	-	+	1.93	2.01	0.98
AT1G33960	AIG1	protein AIG1	+	+	-	1.93	6.98	7.5
AT4G38560	AT4G38560	phospholipase like protein (PEARLI 4)	-	+	-	1.93	2.77	3.05
AT1G15520	ABCG40	ABC transporter G family member 40	+	+	+	1.92	4.76	3.21
AT5G25440	AT5G25440	protein kinase family protein	+	+	-	1.92	3.13	4.1
AT4G13900	RLP50	receptor like protein 50	+	-	-	1.91	2.02	5.17
AT2G37710	RLK	receptor lectin kinase	+	+	+	1.9	2.78	2.66
AT3G16720	ATL2	RING-H2 finger protein ATL2	+	+	+	1.9	2.43	2.27
AT1G62300	WRKY6	WRKY transcription factor 6	+	-	+	1.89	2.9	2.36
AT3G48090	EDS1	enhanced disease susceptibility 1 protein	+	+	+	1.88	4.07	3.58
AT3G18830	PMT5	polyol transporter 5	+	-	-	1.88	1.77	1.41
AT1G02450	NIMIN1	protein NIM1-INTERACTING 1	+	+	+	1.87	4.26	2.65
AT1G73805	SARD1	protein SAR Deficient 1	+	+	+	1.86	3.82	4.15
AT3G02840	AT3G02840	hypothetical protein	+	-	-	1.84	2.88	3.25
AT2G31880	SOBIR1	leucine-rich repeat receptor-like serine/threonine/tyrosine-protein kinase SOBIR1	+	-	-	1.84	3.47	4.15
AT4G04220	RLP46	receptor like protein 46	+	+	+	1.84	2.71	2.93
AT5G63490	AT5G63490	CBS / octicosapeptide/Phox/Bemp1 domain-containing protein	-	+	-	1.83	1.74	1.6
AT2G33380	RD20	caleosin 3	-	+	-	1.82	-0.75	2.16
AT2G19190	FRK1	FLG22-induced receptor-like kinase 1	+	+	+	1.81	4.49	2.61
AT5G38000	AT5G38000	zinc-binding dehydrogenase family protein///zinc-binding dehydrogenase family protein	-	-	+	A	1.86	A

AT3G45860	CRK4	cysteine-rich receptor-like protein kinase 4	+	+	-	1.79	3.33	4.41
AT2G02310	PP2-B6	putative F-box protein PP2-B6	+	-	-	A	2.94	2.68
AT2G46830	CCA1	protein CCA1	-	+	-	1.77	-6.44	4.25
AT2G17040	NAC036	NAC domain containing protein 36	+	-	-	1.77	2.4	3.95
AT1G56650	PAP1	transcription factor MYB75	+	-	-	1.76	-0.7	0.2
AT5G17300	RVE1	myb family transcription factor	-	+	-	1.76	A	4.89
AT3G57260	BGL2	glucan endo-1,3-beta-D-glucosidase	+	+	+	1.75	3.24	6.37
AT2G13790	SERK4	somatic embryogenesis receptor kinase 5///somatic embryogenesis receptor kinase 4	+	-	-	1.74	2.88	2.89
AT2G25735	AT2G25735	hypothetical protein	+	-	+	1.74	1.72	0.42
AT1G67800	AT1G67800	Copine (Calcium-dependent phospholipid-binding protein) family protein	+	+	-	1.73	2.55	2.3
AT1G23710	AT1G23710	hypothetical protein	+	-	+	1.72	2.42	2.79
AT1G03770	RING1B	putative E3 ubiquitin-protein ligase RING1b	-	-	+	A	A	1.22
AT2G39650	AT2G39650	hypothetical protein	+	-	+	1.72	2.37	2.39
AT3G09020	AT3G09020	alpha 1,4-glycosyltransferase family protein	+	+	+	1.71	2.07	1.46
AT2G41010	CAMBP25	calmodulin binding protein 25	-	+	-	1.71	1.89	3.27
AT3G11840	PUB24	E3 ubiquitin-protein ligase PUB24	+	+	+	1.69	2.77	2.85
AT5G60900	RLK1	receptor-like protein kinase 1	+	+	+	1.69	2.72	3.1
AT3G11820	SYP121	syntaxin-121	+	+	+	1.66	2.6	2.12
AT5G39610	NAC6	NAC-domain transcription factor	-	-	+	1.66	1.1	1.74
AT3G09600	RVE8	MYB-like transcription factor	-	+	-	1.66	0.14	4.62
AT3G02000	ROXY1	glutaredoxin-C7	-	+	-	1.66	1.33	0.92
AT5G05190	AT5G05190	hypothetical protein	+	-	+	1.65	2.26	1.52
AT2G23810	TET8	tetraspanin8	+	-	-	1.64	2.64	2.63
AT5G47220	ERF2	ethylene-responsive transcription factor 2	+	-	+	1.64	2.17	0.53
AT3G17790	PAP17	purple acid phosphatase 17	-	-	+	1.62	2.45	3.53
AT5G47910	RBOHD	respiratory burst oxidase-D	+	-	+	1.62	1.54	2.05
AT2G34930	AT2G34930	disease resistance-like protein/LRR domain-containing protein	+	-	-	1.62	0.01	-0.72
AT5G67330	NRAMP4	metal transporter Nramp2	+	-	+	1.62	0.62	0.69
AT4G37150	MES9	methyl esterase 9	+	-	-	1.62	A	2.61
AT1G76040	CPK29	calcium-dependent protein kinase 29	-	+	-	1.61	3.28	3.24
AT1G51890	AT1G51890	probable LRR receptor-like protein kinase	+	+	-	1.6	4.62	3.86
AT3G50950	ZAR1	disease resistance RPP13-like protein 4	+	-	-	1.59	2.95	2.32
AT3G10500	NAC053	NAC domain containing protein 53	-	-	+	1.59	2.68	2.25
AT1G76070	AT1G76070	hypothetical protein	+	-	-	1.58	1.21	1.29
AT3G45640	MPK3	mitogen-activated protein kinase 3	+	+	+	1.58	2.08	1.99
AT1G55690	AT1G55690	Sec14p-like phosphatidylinositol transfer family protein	-	-	+	1.58	1.17	1.2
AT1G52400	BGLU18	beta glucosidase 18	+	-	-	1.58	0.24	-0.08
AT5G58940	CRCK1	calmodulin-binding receptor-like cytoplasmic kinase 1	-	+	+	A	2.22	2.97
AT3G28930	AIG2	avrRpt2-induced protein AIG2	+	+	+	1.56	2.45	1.26
AT5G62520	SRO5	probable inactive poly [ADP-ribose] polymerase SRO5	-	-	+	A	1.82	A
AT2G35930	PUB23	E3 ubiquitin-protein ligase PUB23	+	+	+	1.55	2.29	1.37
AT1G19640	JMT	jasmonic acid carboxyl methyltransferase	-	+	-	1.55	0.28	0.85
AT1G17600	AT1G17600	TIR-NBS-LRR class disease resistance protein	+	+	-	A	3.22	3.2
AT1G10170	NFXL1	NF-X1-type zinc finger protein NFXL1	+	+	+	1.54	1.85	0.85
AT3G19580	ZF2	zinc-finger protein 2	+	-	+	1.53	2.51	2.67
AT5G42050	AT5G42050	DCD (Development and Cell Death) domain protein	+	+	-	1.52	2.49	3.2

AT1G42990	BZIP60	bZIP transcription factor 60	+	+	+	1.52	2.13	2.46
AT5G48380	BIR1	BAK1-interacting receptor-like kinase BIR1	+	+	-	1.52	3.13	2.83
AT4G39830	AT4G39830	putative L-ascorbate oxidase	-	+	-	1.52	5.07	4.74
AT2G46270	GBF3	G-box binding factor 3	-	-	+	1.51	1.95	3.81
AT5G12020	HSP17.6II	class II heat shock protein 17.6	-	-	+	A	A	1.25
AT1G45145	TRX5	thioredoxin H5	+	-	+	1.49	2.89	3.76
AT3G08720	S6K2	serine/threonine protein kinase 2	-	+	-	1.49	2.17	3.3
AT2G02930	GSTF3	glutathione S-transferase F2///glutathione S-transferase F3	+	-	-	1.49	3.06	3.34
AT2G27310	AT2G27310	F-box protein	+	-	+	1.48	2.18	1.39
AT3G09830	AT3G09830	protein kinase family protein	+	+	-	1.48	1.83	2
AT1G50180	AT1G50180	NB-ARC domain-containing disease resistance protein	+	-	-	1.48	A	1.74
AT1G70530	CRK3	cysteine-rich receptor-like protein kinase 3	+	-	-	1.47	1.52	0.14
AT4G24160	AT4G24160	lysophosphatidic acid acyltransferase	+	-	-	1.47	1.83	1.32
AT5G48570	ROF2	peptidyl-prolyl cis-trans isomerase FKBP65	-	-	+	1.46	2.22	0.96
AT5G61890	AT5G61890	ethylene-responsive transcription factor ERF114	+	-	-	1.46	0.8	A
AT1G28370	ERF11	ethylene-responsive transcription factor 11	+	+	-	1.46	1.6	1.82
AT5G02490	Hsp70-2	heat shock protein 70	-	+	+	1.46	4.3	3.27
AT4G02410	LPK1	L-type lectin-like protein kinase 1	+	+	-	1.45	1.66	1.55
AT5G61900	BON1	copine-like protein BONZAI 1	+	+	-	1.45	2.95	2.54
AT4G34150	AT4G34150	calcium-dependent lipid-binding domain-containing protein	+	-	-	1.44	2.28	2.5
AT5G47230	ERF5	ethylene-responsive transcription factor 5	+	-	+	1.44	0.52	-0.74
AT5G45110	NPR3	NPR1-like protein 3	+	+	-	1.44	2.87	2.53
AT1G14360	UTR3	UDP-galactose transporter 3	-	-	+	1.44	2.28	1.7
AT4G11850	PLDGAMMA1	phospholipase D gamma 1	+	-	+	1.43	2.4	1.8
AT4G13510	AMT1;1	ammonium transporter 1;1	+	+	+	1.43	3.36	2.46
AT4G34710	ADC2	arginine decarboxylase 2	-	+	+	1.43	1.17	1.6
AT3G28340	GATL10	putative galacturonosyltransferase-like 10	-	-	+	1.42	2.85	2.46
AT5G46520	VICTR	TIR-NBS-LRR protein VICTR	+	-	-	1.42	2.4	1.87
AT4G31800	WRKY18	WRKY DNA-binding protein 18	+	+	-	1.41	4.18	3.57
AT1G24150	FH4	formin-like protein 4	-	+	-	1.4	2.12	2.22
AT1G66160	CMPG1	U-box domain-containing protein	+	-	+	1.4	0.63	1.8
AT5G16970	AER	2-alkenal reductase	-	-	+	1.4	0.98	0.43
AT5G44070	CAD1	phytochelatin synthase 1	+	+	+	1.39	2.04	1.21
AT3G04210	AT3G04210	TIR-NBS class disease resistance protein	+	-	-	1.39	1.63	1.43
AT5G51190	AT5G51190	ethylene-responsive transcription factor ERF105	+	-	+	1.38	1.06	0.82
AT1G20310	AT1G20310	hypothetical protein	-	+	-	1.38	1.83	1.71
AT5G43580	UPI	Serine protease inhibitor, potato inhibitor I-type family protein	+	-	-	A	A	1.53
AT4G38550	AT4G38550	phospholipase like protein (PEARLI 4) family	+	-	-	1.37	1.88	0.88
AT2G38290	AMT2	ammonium transporter 2	+	+	+	1.37	2.11	1.19
AT1G72930	TIR	toll/interleukin-1 receptor-like protein///Toll-Interleukin-Resistance domain-containing prot	+	-	-	1.37	1.05	1.45
AT1G76970	AT1G76970	Target of Myb protein 1	+	+	-	1.37	2.57	2.66
AT1G17860	AT1G17860	kunitz type trypsin and protease inhibitor domain-containing protein	-	-	+	1.36	1.37	0.48
AT2G02220	PSKR1	phytosulfokin receptor 1	+	-	-	1.36	1.74	1.52
AT5G06960	OBF5	OCS-element binding factor 5	+	+	+	1.35	1.35	2.18
AT1G70690	HWI1	plasmodesmata-located protein 5	+	-	-	1.35	3.05	4.12
AT5G04720	ADR1-L2	ADR1-like 2 protein	+	+	-	1.35	2.52	2.78

AT2G01830	WOL	histidine kinase	+	-	-	1.34	1.48	0.2
AT3G44260	CAF1a	putative CCR4-associated factor 1	+	-	-	1.34	1.85	1.63
AT1G71220	EBS1	UDP-glucose:glycoprotein glucosyltransferase	+	+	-	1.34	2.06	1.42
AT5G09440	EXL4	protein exordium like 4	+	-	-	1.33	0.68	1.69
AT2G38860	YLS5	protease I (pfpI)-like protein YLS5	+	-	-	1.33	2.92	2.1
AT5G22060	J2	chaperone protein dnaJ 2	-	-	+	1.32	2.73	2.37
AT3G02150	PTF1	transcription factor TCP13	+	-	-	1.32	0.9	1.26
AT4G16660	AT4G16660	heat shock protein 70	-	-	+	1.32	2.63	2.01
AT1G19300	PARVUS	putative galacturonosyltransferase-like 1	-	+	-	1.32	2.21	1.66
AT2G15080	RLP19	receptor like protein 19	+	+	+	1.32	1.4	2.07
AT4G23570	SGT1A	phosphatase SGT1a	+	-	-	1.31	2.22	1.96
AT1G74710	EDS16	Isochorismate synthase 1	+	+	+	1.31	3.56	3.64
AT1G22400	UGT85A1	UDP-glycosyltransferase 85A1	-	+	-	1.31	3	2.31
AT4G34390	XLG2	extra-large GTP-binding protein 2	+	+	-	1.31	2.65	2.72
AT1G48000	MYB112	myb domain protein 112	-	+	-	A	A	2.58
AT4G37640	ACA2	calcium-transporting ATPase 2	+	+	-	1.31	2.74	2.35
AT5G22250	CAF1b	CCR4-associated factor 1B	+	-	+	1.31	2.09	1.27
AT1G03850	GRXS13	glutaredoxin ATGRXS13	+	-	-	1.3	3.2	2.06
AT5G61560	AT5G61560	U-box domain-containing protein 51	+	-	+	1.29	2.01	1.59
AT2G30770	CYP71A13	cytochrome P450, family 71, subfamily A, polypeptide 13	+	+	+	1.29	5.45	4.97
AT5G03210	DIP2	hypothetical protein	+	-	-	1.29	1.39	1.77
AT4G36150	AT4G36150	TIR-NBS-LRR class disease resistance protein	+	+	+	1.29	2.2	1.63
AT5G26030	FC1	ferrochelatase 1	+	-	+	1.28	1.63	1.73
AT5G47070	AT5G47070	protein kinase family protein	-	+	-	1.28	2.11	2.15
AT5G42650	AOS	allene oxide synthase	+	+	+	1.28	-0.79	-1.58
AT2G47730	GSTF8	glutathione S-transferase phi 8	+	-	-	1.28	2.19	1.48
AT3G02800	PFA-DSP3	atypical dual-specificity phosphatase	-	-	+	1.27	1.68	1.17
AT3G46710	AT3G46710	putative disease resistance RPP13-like protein 2	+	-	-	A	A	1.09
AT4G31500	CYP83B1	cytochrome P450 83B1	+	-	-	1.27	1.92	0.87
AT5G10760	AT5G10760	aspartyl protease family protein	+	-	-	1.27	2.9	3.73
AT2G22330	CYP79B3	tryptophan N-hydroxylase 2	+	-	-	1.25	0.64	0.29
AT3G46600	AT3G46600	scarecrow-like protein 30	+	-	+	1.23	1.8	0.88
AT4G23130	CRK5	cysteine-rich receptor-like protein kinase 5	+	+	-	1.23	1.26	0.63
AT4G26070	MEK1	mitogen-activated protein kinase kinase 1	+	+	+	1.22	2.12	1.7
AT2G34940	VSR5	vacuolar-sorting receptor 5	-	+	-	1.21	2.24	2.34
AT1G07000	EXO70B2	exocyst subunit exo70 family protein B2	+	+	-	1.21	2.64	2.93
AT3G54640	TSA1	tryptophan synthase alpha chain	+	+	+	1.21	1.48	0.57
AT4G26090	RPS2	disease resistance protein RPS2	+	+	+	1.2	2.02	1.36
AT5G53120	SPDS3	Spermine synthase	+	-	-	1.2	1.33	2.21
AT3G17700	CNBT1	cyclic nucleotide-binding transporter 1	+	+	+	1.2	2.58	2.04
AT1G80820	CCR2	cinnamoyl-CoA reductase	+	-	-	1.2	0.66	2.53
AT1G77920	TGA7	transcription factor TGA7	+	-	-	1.19	1.28	0.93
AT1G64070	RLM1	TIR-NBS-LRR class disease resistance protein	+	-	-	A	1.3	A
AT5G06870	PGIP2	polygalacturonase inhibitor 2	+	+	+	1.19	-0.45	-0.13
AT3G20470	GRP5	glycine-rich protein 5	-	+	-	1.19	0.01	-0.88
AT5G45000	AT5G45000	TIR-NBS-LRR class disease resistance protein	+	-	-	1.18	3.66	2.89

AT1G56540	AT1G56540	TIR-NBS-LRR class disease resistance protein	+	-	-	A	A	2.37
AT2G04450	NUDT6	nudix hydrolase 6	+	+	+	1.18	5.6	5.49
AT1G19670	CLH1	chlorophyllase 1	+	+	+	1.18	-0.05	-0.11
AT4G38540	AT4G38540	FAD/NAD(P)-binding oxidoreductase family protein	+	-	-	1.18	2.77	2.09
AT1G08450	CRT3	calreticulin-3	+	+	-	1.17	2.54	3.43
AT1G08930	ERD6	sugar transporter ERD6	+	-	-	1.17	1.71	1.08
AT1G79680	WAKL10	wall-associated receptor kinase-like 10	+	+	-	1.16	2.28	1.07
AT3G10040	AT3G10040	sequence-specific DNA binding transcription factor	-	+	+	1.16	1.05	0.06
AT1G32640	MYC2	transcription factor MYC2	+	+	+	1.16	0.38	1.21
AT5G63970	RGLG3	ring domain ligase 3	+	-	-	1.15	0.85	0.77
AT1G74440	AT1G74440	hypothetical protein	+	-	-	1.14	2.04	2.67
AT3G09610		MYB-like transcription factor	-	+	-	1.14	-2.27	3.55
AT4G39950	CYP79B2	tryptophan N-monooxygenase 1	+	-	-	1.14	1.73	1.05
AT3G57330	ACA11	putative calcium-transporting ATPase 11	+	-	-	1.13	1.57	1.62
AT1G25390	AT1G25390	protein kinase-like protein	+	-	-	1.13	1.76	1.4
AT1G14200	AT1G14200	RING finger domain-containing protein	-	-	+	1.13	1.42	1.45
AT1G59870	PEN3	ABC transporter G family member 36	+	+	+	1.13	1.56	0.65
AT1G61560	MLO6	MLO-like protein 6	+	-	-	1.12	0.5	1.04
AT4G11190	AT4G11190	disease resistance-responsive, dirigent domain-containing protein	+	-	-	1.12	-0.25	-1.12
AT1G04980	PDIL2-2	protein disulfide-isomerase like 2-2	-	-	+	1.12	2.91	2.44
AT4G36500	AT4G36500	hypothetical protein	+	+	-	1.12	0.96	0.42
AT5G24660	LSU2	response to low sulfur 2	+	-	-	1.12	0.31	2.12
AT5G06320	NHL3	NDR1/HIN1-Like protein 3	+	-	-	1.12	1.77	2.21
AT1G54840	AT1G54840	alpha-crystallin domain of heat shock protein-containing protein	-	-	+	1.12	1.69	A
AT2G30020	AT2G30020	putative protein phosphatase 2C 53///putative protein phosphatase 2C-like protein 45///putati	+	-	-	1.12	1.82	1.98
AT3G50260	CEJ1	ethylene-responsive transcription factor ERF011	+	-	+	1.12	2.32	1.36
AT4G04500	CRK37	cysteine-rich receptor-like protein kinase 37	-	+	-	1.11	4.36	5.58
AT5G62570	AT5G62570	calmodulin binding protein-like protein	+	-	-	1.11	0.9	2.12
AT1G77500	AT1G77500	hypothetical protein	+	-	-	1.11	1.97	1.5
AT3G16890	PPR40	pentatricopeptide repeat-containing protein	-	-	+	1.1	A	A
AT4G19660	NPR4	NPR1-like protein 4	+	+	-	1.1	1.64	1.16
AT4G28400	AT4G28400	putative protein phosphatase 2C 58	+	-	-	1.1	1.4	2.38
AT5G18750	AT5G18750	DNAJ heat shock N-terminal domain-containing protein	-	-	+	1.1	0.9	0.19
AT2G41180	SIB2	VQ motif-containing protein	+	-	-	1.1	1.59	0.92
AT5G49520	WRKY48	putative WRKY transcription factor 48	+	-	+	A	1.66	2.71
AT5G09590	MTHSC70-2	mitochondrial HSO70 2	-	-	+	1.09	1.86	0.68
AT2G39660	BIK1	serine/threonine-protein kinase BIK1	+	+	+	1.09	1.65	1.69
AT1G57560	MYB50	myb domain protein 50	-	+	-	1.09	3.87	2.76
AT1G19230	AT1G19230	riboflavin synthase-like superfamily protein	+	-	-	1.09	A	0.38
AT4G08480	MAPKKK9	mitogen-activated protein kinase kinase kinase 9	+	-	-	1.09	1.28	1.16
AT1G18380		probable serine/threonine-protein kinase	+	-	+	1.08	1.6	0.61
AT3G23240	ERF1	ethylene-responsive transcription factor 1B	+	-	-	1.08	1.54	1.19
AT1G51620	AT1G51620	protein kinase superfamily protein	+	-	+	A	2.2	2.71
AT5G48600	SMC3	structural maintenance of chromosomes protein 4	+	-	-	1.08	1.7	0.69
AT4G31750	WIN2	putative protein phosphatase 2C 59	+	-	-	1.08	1.37	1.84
AT4G15430	AT4G15430	ERD (early-responsive to dehydration stress) family protein	-	+	-	1.08	0.17	3.06

AT3G05200	ATL6	E3 ubiquitin-protein ligase ATL6	+	-	+	1.07	1.61	1.19
AT1G69260	AFP1	ABI five binding protein	-	+	-	1.07	1.27	3.36
AT1G75280	AT1G75280	putative Isoflavone reductase	-	-	+	1.07	-0.72	-0.53
AT3G09440	AT3G09440	heat shock protein 70-3	-	-	+	1.07	2.42	1.46
AT4G17230	SCL13	protein scarecrow-like 13	+	+	+	1.07	1.63	0.78
AT1G07630	PLL5	putative protein phosphatase 2C	+	+	-	1.07	1.27	1.53
AT2G26530	AR781	hypothetical protein	-	+	-	1.07	0.64	0.12
AT2G17850	AT2G17850	rhodanese-like domain-containing protein 17	-	+	+	1.06	A	A
AT2G04430	NUDT5	nudix hydrolase 5	+	+	-	1.06	4.38	4.45
AT2G16060	HB1	non-symbiotic hemoglobin 1	-	+	+	1.06	4.4	2.3
AT5G13190	GILP	GSH-induced LITAF domain protein	-	+	-	1.06	2.09	2.04
AT5G17990	TRP1	anthranilate phosphoribosyltransferase	+	-	-	1.06	1.58	1.16
AT4G18010	IP5PII	Type I inositol-1,4,5-trisphosphate 5-phosphatase 2	+	-	-	1.05	0.89	1.62
AT1G18390	AT1G18390	probable serine/threonine-protein kinase	+	-	+	1.05	2.33	0.55
AT1G73730	EIL3	ethylene insensitive 3-like 3 protein	+	+	+	1.05	0.52	1.08
AT4G09570	CPK4	calcium-dependent protein kinase 4	+	+	-	1.05	1.9	1.41
AT2G30550	AT2G30550	phospholipase A1-Igamma2	+	+	-	1.05	2.77	2.71
AT5G26860	LON1	lon protease 1	-	-	+	1.05	1.72	0.53
AT2G40270	AT2G40270	Protein kinase family protein	+	+	-	1.04	1.17	2.26
AT4G25390	AT4G25390	receptor-like serine/threonine-protein kinase	-	+	-	1.04	1.45	1.65
AT3G02550	LBD41	LOB domain-containing protein 41	-	+	+	1.04	1.48	-0.14
AT2G17740	AT2G17740	cysteine/histidine-rich C1 domain-containing protein	+	-	+	1.04	1.55	A
AT5G17760	AT5G17760	P-loop containing nucleoside triphosphate hydrolases superfamily protein	-	+	-	1.03	4.64	4.89
AT1G12210	RFL1	disease resistance protein RFL1	+	-	-	A	2.98	A
AT2G17720	P4H5	prolyl 4-hydroxylase 5	+	-	+	1.02	2.02	1.72
AT4G24190	SHD	HSP90-like protein GRP94	-	-	+	1.02	2.19	1.88
AT3G07720	AT3G07720	galactose oxidase/kelch repeat-containing protein	-	+	-	1.02	1.88	2.33
AT1G54100	ALDH7B4	aldehyde dehydrogenase 7B4	-	-	+	1.02	0.7	0.91
AT3G09350	Fes1A	protein Fes1A	-	-	+	1	1.61	1.18
AT5G59550	RDUF2	ABA- and drought-induced RING-DUF1117 protein	+	-	+	1	1.78	1.39
AT2G04400	AT2G04400	indole-3-glycerol phosphate synthase	+	-	-	0.99	2.08	0.77
AT2G34720	NF-YA4	nuclear transcription factor Y subunit A-4	-	+	-	0.98	-0.73	1.64
AT3G45620	AT3G45620	DWD motif protein	-	+	-	0.98	2.35	2.5
AT1G64280	NPR1	Regulatory protein NPR1	+	+	+	0.98	1.35	1.34
AT1G64610	AT1G64610	WD40 domain-containing protein	+	+	+	0.98	2.11	2.14
AT4G14220	RHF1A	E3 ubiquitin-protein ligase RHF1A	+	+	+	0.98	1.59	1.97
AT2G37940	AtIPCS2	inositol phosphorylceramide synthase 2	+	-	-	0.97	1.67	1.22
AT1G74100	SOT16	sulfotransferase 16	+	-	-	0.96	1.12	0.64
AT3G19970	AT3G19970	hypothetical protein	+	+	-	0.96	0.96	2.71
AT1G07520	AT1G07520	GRAS family transcription factor	+	-	+	0.96	1.3	0.94
AT3G24500	MBF1C	multiprotein-bridging factor 1c	-	-	+	0.96	2.09	2.6
AT5G39020	AT5G39020	putative receptor-like protein kinase	+	+	+	0.95	1.5	0.91
AT2G22300	SR1	calmodulin-binding transcription activator 3	+	-	-	0.95	1.43	1.21
AT3G21630	CERK1	chitin elicitor receptor kinase 1	+	+	+	0.95	1.62	1.3
AT3G01320	SNL1	paired amphipathic helix protein Sin3-like 1	-	-	+	0.95	1.9	1.29
AT5G58120	AT5G58120	TIR-NBS-LRR class disease resistance protein	+	-	-	0.95	1.83	3.64

AT2G25000	WRKY60	putative WRKY transcription factor 60	+	+	+	0.94	1.76	4.02
AT4G23480		PLAC8 family protein	+	+	-	0.94	2.1	2.43
AT2G47190	MYB2	R2R3 MYB DNA binding domain transcription factor	-	+	-	A	A	1.59
AT1G33560	ADR1	disease resistance protein ADR1	+	-	-	0.94	0.96	1.8
AT2G25140	CLPB4	casein lytic proteinase B4	-	-	+	0.92	1.67	1.26
AT3G46620	RDUF1	C3H4 type zinc finger protein	+	-	+	0.92	1.4	1.65
AT2G47180	GolS1	galactinol synthase 1	-	-	+	0.92	-2.44	0.81
AT4G16760	ACX1	peroxisomal acyl-coenzyme A oxidase 1	+	+	-	0.92	0.79	1.62
AT1G05010	EFE	1-aminocyclopropane-1-carboxylate oxidase	+	+	+	0.92	1.49	0.91
AT1G75020	LPAT4	lysophosphatidyl acyltransferase 4	-	+	-	0.91	1.25	1.84
AT2G27660	AT2G27660	cysteine/histidine-rich C1 domain-containing protein	+	-	+	A	1.24	3.43
AT2G16720	MYB7	myb domain protein 7	-	+	-	0.91	1	2.23
AT5G10380	RING1	E3 ubiquitin-protein ligase RING1	+	+	+	0.91	1.46	3.14
AT5G35320	AT5G35320	hypothetical protein	-	-	+	0.91	1.25	1.58
AT1G53350	AT1G53350	putative disease resistance RPP8-like protein	+	-	-	A	1.66	1.07
AT4G26850	VTC2	GDP-L-galactose phosphorylase 1	+	-	-	0.9	-1.07	1.95
AT3G44630	AT3G44630	TIR-NBS-LRR class disease resistance protein	+	-	-	0.89	1.21	2.16
AT1G28190	AT1G28190	hypothetical protein	-	+	-	0.89	1.45	0.81
AT5G46330	FLS2	LRR receptor-like serine/threonine-protein kinase FLS2	+	-	-	0.89	1.32	-0.69
AT1G51820	AT1G51820	putative LRR receptor-like serine/threonine protein kinase	+	-	+	0.89	1.15	0.55
AT2G16900	AT2G16900	phospholipase-like protein (PEARLI 4) family	+	-	+	0.88	1.63	1.39
AT5G24090	CHIA	chitinase A	-	-	+	A	1.33	1.69
AT1G15890	AT1G15890	CC-NBS-LRR class disease resistance protein	+	-	-	0.88	1.15	0.09
AT5G19875	AT5G19875	hypothetical protein	-	-	+	0.88	1.06	3.43
AT5G51630	AT5G51630	TIR-NBS-LRR class disease resistance protein	+	-	-	A	2.05	2.25
AT3G04220	AT3G04220	TIR-NBS-LRR class disease resistance protein	+	-	-	0.87	0.98	1.46
AT5G61010	EXO70E2	exocyst subunit exo70 family protein E2	-	+	-	0.87	1.6	2.16
AT5G61210	SNAP33	soluble N-ethylmaleimide-sensitive factor adaptor protein 33	+	+	+	0.86	1.86	2.03
AT1G13750	AT1G13750	Purple acid phosphatases superfamily protein	+	-	-	0.86	1.9	1.42
AT3G20410	CPK9	calmodulin-domain protein kinase 9	+	+	-	0.86	1.24	1.07
AT3G59700	HLECRK	lectin-receptor kinase	+	-	-	0.85	2.32	1.74
AT1G34750	AT1G34750	putative protein phosphatase 2C 10	+	-	-	0.85	2.38	2.65
AT4G04920	SFR6	mediator of RNA polymerase II transcription subunit 16	-	-	+	0.85	1.87	1.5
AT5G12400	AT5G12400	PHD-finger and DNA binding domain-containing protein	-	-	+	0.84	1.69	0.94
AT5G43910	AT5G43910	pfkB-like carbohydrate kinase family protein	-	+	-	0.84	3.13	3.72
AT5G56030	HSP81-2	heat shock protein 81-2	+	-	-	0.83	1.52	0.64
AT2G11520	CRCK3	calmodulin-binding receptor-like cytoplasmic kinase 3	+	+	-	0.83	1.48	1.73
AT3G54960	PDIL1-3	protein PDI-like 1-3	-	-	+	0.83	2	2.04
AT5G66900	AT5G66900	CC-NBS-LRR class disease resistance protein///CC-NBS-LRR class disease resistance protein	+	-	-	0.83	1.56	1.23
AT4G23450	AIRP1	C3H2C3-type RING E3 Ub ligase	-	-	+	0.83	1.79	2.32
AT2G36470	AT2G36470	hypothetical protein	+	+	+	0.82	2.3	-0.11
AT5G05730	ASA1	anthranilate synthase component I-1	+	+	+	0.81	1.38	0.15
AT1G18890	CDPK1	calcium-dependent protein kinase 1	+	+	-	0.81	1.72	2.24
AT1G72300	PSY1R	tyrosine-sulfated glycopeptide receptor 1	+	-	-	0.8	-0.14	1.02
AT1G77510	PDIL1-2	protein disulfide-isomerase 2	-	-	+	0.8	2.54	2.45
AT5G49570	PNG1	peptide-N(4)-(N-acetyl-beta-glucosaminyl)asparagine amidase	-	+	-	0.79	1.76	2

AT5G46490	AT5G46490	TIR-NBS-LRR class disease resistance protein///TIR-NBS-LRR class disease resistance protein	+	-	-	0.79	0.66	1.15
AT1G14790	RDR1	RNA-dependent RNA polymerase 1	+	+	+	0.79	1.54	1.75
AT1G31280	AGO2	Argonaute family protein	+	-	-	0.78	1.99	1.32
AT3G59660	AT3G59660	C2 domain and GRAM domain-containing protein	+	+	-	0.78	1.68	1.76
AT5G03630	ATMDAR2	monodehydroascorbate reductase (NADH)	-	-	+	0.78	1.23	1.08
AT3G62600	ATERDJ3B	DNAJ heat shock family protein	-	-	+	0.77	1.96	1.86
AT5G61790	CNX1	calnexin 1	-	-	+	0.77	1.71	1.22
AT5G67340	AT5G67340	ARM repeat superfamily protein	+	+	-	0.76	2.73	2.73
AT1G64060	RBOH F	respiratory burst oxidase	+	+	+	0.76	1.15	1.73
AT1G70520	CRK2	cysteine-rich receptor-like protein kinase 2	-	-	+	0.76	1.15	0.37
AT2G30870	GSTF10	glutathione S-transferase PHI 10	+	-	-	0.76	1.21	-0.27
AT1G50740	AT1G50740	transmembrane protein 14C	+	-	-	0.75	1.72	1.89
AT1G30620	MUR4	UDP-arabinose 4-epimerase	+	-	-	0.75	2.15	1.28
AT3G25230	ROF1	rotamase FKBP 1	-	-	+	0.75	1.16	0.22
AT4G03960	PFA-DSP4	atypical dual-specificity phosphatase 4	+	-	-	0.75	1.35	1.27
AT3G07040	RPM1	disease resistance protein RPM1	+	-	-	0.74	1.14	0.72
AT5G27830	AT5G27830	hypothetical protein	-	-	+	0.74	1.68	1.95
AT2G27170	TTN7	structural maintenance of chromosome 3	+	-	-	0.74	1.07	A
AT1G70140	FH8	formin-like protein 8	+	-	+	0.74	1.9	1.66
AT3G18690	MKS1	MAP kinase substrate 1	+	-	-	0.74	1.23	1.04
AT3G15210	ERF4	ethylene-responsive transcription factor 4	+	-	+	0.73	1.14	1.58
AT1G31130	AT1G31130	hypothetical protein	+	-	-	0.73	1.01	1.6
AT1G21270	WAK2	wall-associated receptor kinase 2	-	+	-	0.72	1.26	0.94
AT4G12470	AZI1	azelaic acid induced 1	+	+	-	0.71	2.63	0.12
AT1G63750	AT1G63750	TIR-NBS-LRR class disease resistance protein	+	-	-	A	1.29	2.14
AT3G49120	PRXCB	peroxidase 34///peroxidase 33	+	-	+	0.7	2.06	2.02
AT1G63740	AT1G63740	TIR-NBS-LRR class disease resistance protein	+	-	-	0.7	1.25	0.38
AT4G08770	Prx37	putative apoplastic peroxidase Prx37	+	-	-	A	2.07	2.29
AT5G10190	AT5G10190	major facilitator protein	+	-	-	0.7	1.18	1.35
AT1G76930	EXT4	extensin 4	-	+	-	0.7	0.09	1.26
AT2G05940	RIPK	RPM1-induced protein kinase	+	+	-	0.7	1.81	2.09
AT3G46930	AT3G46930	protein kinase family protein	-	-	+	A	1.85	1.98
AT3G05650	RLP32	receptor like protein 32	+	+	+	0.69	2.66	2.87
AT2G17290	CPK6	Calcium-dependent protein kinase family protein	+	+	-	0.68	1.87	2.37
AT5G05410	DREB2A	dehydration-responsive element-binding protein 2A	-	-	+	0.68	3.14	0.84
AT4G17070	AT4G17070	peptidyl-prolyl cis-trans isomerase	-	-	+	0.68	1.73	2.32
AT4G12120	SEC1B	protein transport sec1b	+	-	-	0.68	1.89	1.37
AT3G28940	AT3G28940	AIG2-like protein	+	-	-	0.68	1.12	0.75
AT3G50970	LTI30	dehydrin Xero 2	+	-	-	0.68	0.54	-1.18
AT3G57530	CPK32	calcium-dependent protein kinase 32	+	+	-	0.68	1.28	1.73
AT3G45040	AT3G45040	putative dolichol kinase	+	+	-	0.68	1.51	1.61
AT2G44180	MAP2A	methionine aminopeptidase 2A	+	-	-	0.67	2.01	2.19
AT1G72950	AT1G72950	TIR-NBS class of disease resistance protein	+	-	-	A	A	1.76
AT4G23850	LACS4	long chain acyl-CoA synthetase 4	+	-	-	0.67	1.18	1.81
AT4G01010	CNGC13	cyclic nucleotide-gated channel 13	+	+	-	0.67	1.7	2.25
AT3G21220	MKK5	mitogen-activated protein kinase kinase 5	+	-	-	0.67	1.14	1.64

AT1G56340	CRT1a	calreticulin-1	-	-	+	0.67	1.17	0.67
AT5G66070	AT5G66070	RING/U-box superfamily protein	+	-	+	0.67	1.32	0.44
AT5G60360	ALP	thiol protease aleurain	-	-	+	0.67	0.74	1.44
AT3G63420	GG1	Ggamma-subunit 1	+	-	-	0.66	1.39	1.06
AT4G29520	AT4G29520	hypothetical protein	-	-	+	0.66	1.84	1.63
AT3G46530	RPP13	disease resistance protein RPP13	+	-	-	0.66	2.19	1.71
AT2G17220	Kin3	putative serine/threonine-specific protein kinase kin3	+	+	-	0.65	1.79	1.98
AT1G12200	FMO	putative flavin monooxygenase.	+	-	-	0.65	2.04	1.06
AT3G01080	WRKY58	WRKY DNA-binding protein 58	+	-	-	A	4.13	4.34
AT3G63010	GID1B	putative gibberellin receptor GID1L2	+	+	+	A	0.97	2.34
AT1G66080	AT1G66080	hypothetical protein	-	-	+	0.65	1.15	1.34
AT1G56510	WRR4	TIR-NB-LRR disease resistance protein	+	-	-	0.65	1.29	2.28
AT5G55070	AT5G55070	dihydrolipoyllysine-residue succinyltransferase component of 2-oxoglutarate dehydrogenase com	-	-	+	0.64	0.93	1.11
AT5G55450	AT5G55450	bifunctional inhibitor/lipid-transfer protein/seed storage 2S albumin-like protein	+	+	+	0.64	2.98	2.89
AT1G59124	AT1G59124	putative disease resistance protein///putative disease resistance protein RDL5	+	-	-	0.63	1.14	1.24
AT5G55310	TOP1BETA	DNA topoisomerase 1 beta	+	-	-	0.63	0.86	1.04
AT2G02810	UTR1	UDP-galactose transporter 1	-	-	+	0.63	1.51	1.15
AT1G72890	AT1G72890	TIR-NBS class of disease resistance protein	+	-	-	0.63	0.74	1.24
AT4G08180	ORP1C	OSBP(oxysterol binding protein)-related protein 1C	+	+	-	0.62	1.05	0.8
AT5G07340	AT5G07340	calnexin2	-	-	+	0.62	1.24	0.99
AT4G33300	ADR1-L1	putative disease resistance protein ADR1-like 1	+	-	-	0.62	1.54	1.72
AT5G38710	AT5G38710	proline dehydrogenase 2	-	+	-	0.61	1.77	1.1
AT3G16050	PDX1.2	putative pyridoxal biosynthesis protein PDX1.2	-	-	+	0.61	2.03	2.01
AT1G13340	AT1G13340	Regulator of Vps4 activity in the MVB pathway protein	+	+	+	0.61	2.61	4.04
AT1G65870	AT1G65870	Disease resistance-responsive (dirigent-like protein) family protein	+	-	-	0.61	0.27	2.91
AT1G54040	ESP	epithiospecifier protein	+	-	-	0.61	0.02	-1
AT3G10640	VPS60.1	vacuolar protein sorting protein 60.1	+	+	-	0.61	1.2	1.24
AT1G69270	RPK1	receptor-like protein kinase 1	+	+	+	0.6	1.62	1.65
AT3G49530	NAC062	NAC domain-containing protein 62	+	-	+	0.6	1.99	1.72
AT5G27520	PNC2	peroxisomal adenine nucleotide carrier 2	-	+	-	0.59	1.07	1.26
AT3G51130	AT3G51130	hypothetical protein	-	+	-	0.59	1.03	1.38
AT2G04795	AT2G04795	hypothetical protein	-	-	+	0.59	0.01	2.73
AT2G16500	ADC1	arginine decarboxylase 1	-	-	+	0.59	0.02	1.13
AT3G13050	NiaP	nicotinate transporter	-	+	-	0.59	0.97	1.05
AT5G03160	P58IPK	mamallian P58IPK-like protein	-	-	+	0.58	1.38	1.34
AT1G78380	GSTU19	glutathione S-transferase TAU 19	-	-	+	0.57	1.07	0.59
AT4G37560	AT4G37560	acetamidase/formamidase family protein	+	-	-	0.57	-0.07	1.5
AT5G40910	AT5G40910	TIR-NBS-LRR class disease resistance protein	+	-	-	0.57	1.02	0.91
AT1G11310	MLO2	MLO-like protein 2	+	+	+	0.57	1.73	1.24
AT5G46350	WRKY8	putative WRKY transcription factor 8	+	-	+	0.57	1.76	2.36
AT1G16670	AT1G16670	kinase domain-containing protein	+	+	-	0.57	2.03	1.5
AT5G35735	AT5G35735	putative auxin-responsive protein	+	+	-	0.57	1.94	1.04
AT1G44414	AT1G44414	hypothetical protein	-	-	+	A	1.68	A
AT1G12290	AT1G12290	CC-NBS-LRR class disease resistance protein	+	-	-	A	1.58	2.65
AT1G33600	AT1G33600	leucine-rich repeat-containing protein	+	+	+	0.56	0.62	-1.14
AT4G17880	MYC4	transcription factor MYC4	+	-	-	0.56	-0.61	1.52

AT1G76770	AT1G76770	alpha-crystallin domain of heat shock protein-containing protein	-	-	+	A	1.39	A
AT1G70000	AT1G70000	myb-like transcription factor	-	+	-	0.55	0.36	2.57
AT5G36930	AT5G36930	TIR-NBS-LRR class disease resistance protein	+	-	-	0.55	1.76	1.36
AT1G16540	ABA3	molybdenum cofactor sulfurase	+	+	+	0.55	1.38	1.21
AT4G37460	SRFR1	suppressor of RPS4-RLD 1	+	-	-	0.54	0.07	1.15
AT4G23280	CRK20	putative cysteine-rich receptor-like protein kinase 20	+	+	-	0.54	1.33	0.93
AT3G28210	PMZ	zinc finger (AN1-like) family protein	-	+	-	0.53	3.55	2.14
AT1G73650	AT1G73650	hypothetical protein	+	-	-	0.53	0.35	1.76
AT3G44190	AT3G44190	FAD/NAD(P)-binding oxidoreductase family protein	-	-	+	0.53	1.43	0.93
AT5G22690	AT5G22690	TIR-NBS-LRR class disease resistance protein	+	-	-	0.52	0.42	1.06
AT2G32920	PDIL2-3	protein disulfide-isomerase like 2-3	-	-	+	0.52	1.26	0.89
AT1G22070	TGA3	transcription factor TGA3	+	+	+	0.52	1.38	-0.15
AT1G17610	CHS1	disease resistance protein (TIR-NBS class)	+	+	+	0.52	1.37	1.52
AT5G46510	VICTL	TIR-NBS-LRR class disease resistance protein	+	-	-	0.52	1.13	0.24
AT3G11220	ELO1	elongator complex protein 4	-	-	+	0.52	1.02	1.07
AT4G12000	AT4G12000	SNARE associated Golgi family protein	-	-	+	0.51	-0.06	2.07
AT3G51920	CAM9	calmodulin-like protein	+	-	-	0.51	-0.24	1.8
AT1G62740	Hop2	carboxylate clamp-tetratricopeptide repeat protein	-	-	+	0.5	1.34	0.76
AT1G34420	AT1G34420	leucine-rich repeat transmembrane protein kinase-like protein	+	+	-	A	2.1	2.28
AT3G49350	AT3G49350	RabGAP/TBC domain-containing protein	+	+	-	0.5	0.97	1.07
AT3G60690	AT3G60690	SAUR-like auxin-responsive protein	-	+	-	0.5	0.44	1.56
AT3G52800	AT3G52800	zinc finger A20 and AN1 domain-containing stress-associated protein 6	+	-	+	0.49	1.1	2.25
AT3G23990	HSP60	heat shock protein 60	-	-	+	0.49	1.29	0.06
AT1G27330	AT1G27330	ribosome associated membrane protein RAMP4///ribosome associated membrane protein RAMP4	-	-	+	0.49	0.93	1.01
AT2G46500	PI4K GAMMA	phosphoinositide 4-kinase gamma 4	+	+	+	0.49	1.23	1.32
AT4G02550	AT4G02550	hypothetical protein	+	-	-	0.49	1.26	0.01
AT4G12010	AT4G12010	TIR-NBS-LRR class disease resistance protein	+	-	-	0.48	1.39	0.89
AT2G46510	AIB	transcription factor ABA-INDUCIBLE bHLH-TYPE	-	+	-	0.48	1.06	1.39
AT2G13780	SERK4	somatic embryogenesis receptor kinase 4	+	-	-	A	3.04	A
AT4G34000	ABF3	abscisic acid responsive elements-binding factor 3	-	+	+	0.48	-0.38	1.97
AT2G23320	WRKY15	WRKY DNA-binding protein 15	+	-	+	0.48	1.01	1.3
AT5G53400	BOB1	protein BOBBER 1	-	-	+	0.48	1.22	0.7
AT4G08500	MEKK1	mitogen-activated protein kinase kinase kinase 1	+	+	-	0.47	1.05	1.27
AT5G07460	PMSR2	peptide methionine sulfoxide reductase A2	-	-	+	0.47	-0.15	-1.1
AT5G45260	RRS1	disease resistance protein RRS1	+	-	-	0.46	1.03	0.58
AT1G09210	CRT1b	calreticulin 1b	-	-	+	0.46	0.97	1.13
AT5G21090	AT5G21090	leucine-rich repeat-containing protein	-	-	+	0.46	0.75	1.26
AT1G77120	ADH1	alcohol dehydrogenase 1	-	+	+	0.46	2.87	0.97
AT5G45250	RPS4	TIR-NBS-LRR class disease resistance protein	+	-	-	0.45	1.49	1.19
AT3G46510	PUB13	U-box domain-containing protein 13	+	-	-	0.44	0.68	1.01
AT3G04710	TPR10	carboxylate clamp-tetratricopeptide repeat protein	-	-	+	0.44	1.07	0.15
AT4G00550	DGD2	digalactosyldiacylglycerol synthase 2	+	+	-	0.44	1.24	1.32
AT1G15430	AT1G15430	hypothetical protein	+	+	-	0.44	1.32	1.17
AT1G63460	GPX8	glutathione peroxidase 8	-	-	+	0.43	1.25	1.43
AT2G25110	SDF2	stromal cell-derived factor 2-like protein	+	-	+	0.43	1.35	1.27
AT5G37480	AT5G37480	hypothetical protein	+	+	-	0.43	0.84	1.24

AT4G26970	ACO2	aconitase 2	-	-	+	0.43	1.16	0.81
AT1G65040	Hrd1B	HRD1-like protein	-	-	+	0.42	1.27	0.83
AT5G47250	AT5G47250	putative disease resistance protein	+	-	-	0.42	1.4	1.39
AT4G13810	RLP47	receptor like protein 47	+	-	-	0.42	-0.35	1.42
AT2G42540	COR15A	cold-regulated protein 15a	+	+	+	0.41	0.14	-4.46
AT3G02885	GASA5	gibberellin-regulated protein 5	-	+	-	0.4	-0.6	-1.03
AT5G67480	BT4	BTB and TAZ domain protein 4	-	+	+	0.4	1.58	-1.4
AT1G54320	AT1G54320	ALA-interacting subunit 3	-	+	-	0.4	0.83	1.02
AT2G26300	GP ALPHA 1	guanine nucleotide-binding protein alpha-1 subunit	-	+	+	0.4	1.08	1.14
AT1G58842		putative disease resistance protein RDL6/RF9///putative disease resistance protein RDL6/RF9	+	-	-	0.39	0.9	1.18
AT1G52890	NAC019	NAC domain-containing protein 19	-	+	-	A	1.46	1.68
AT5G17880	CSA1	TIR-NBS-LRR class disease resistance protein	+	-	-	0.38	1.2	1.06
AT1G29690	CAD1	MAC/Perforin domain-containing protein	+	+	+	0.38	1.52	1.25
AT1G05690	BT3	BTB and TAZ domain protein 3	-	+	+	0.38	-0.58	1.09
AT5G20320	DCL4	dicer-like protein 4	+	-	-	0.38	0.83	1.13
AT4G29810	MKK2	mitogen-activated protein kinase kinase 2	+	+	+	0.38	1.66	1.84
AT5G40010	AATP1	AAA-ATPase 1	-	+	+	A	2.52	1.76
AT4G19510	AT4G19510	TIR-NBS-LRR class disease resistance protein	+	-	-	0.37	0.31	1.24
AT1G57830	AT1G57830	Toll-Interleukin-Resistance domain-containing protein	+	-	-	0.37	-1.93	-0.17
AT2G22240	MIPS2	myo-inositol-1-phosphate synthase 2	+	-	+	0.36	-2.51	0.88
AT4G32770	VTE1	tocopherol cyclase	+	-	+	0.36	-1.59	-0.08
AT4G11260	SGT1B	phosphatase SGT1b	+	+	+	0.35	0.87	1.13
AT2G43510	TI1	defensin-like protein 195	+	-	-	0.34	0.3	2.51
AT1G62730	AT1G62730	isoprenoid biosynthesis enzyme domain-containing protein	-	-	+	0.34	1.25	1.03
AT3G11660	NHL1	NDR1/HIN1-Like protein 1	+	-	-	0.34	0.07	1.31
AT1G62630	AT1G62630	CC-NBS-LRR class disease resistance protein///CC-NBS-LRR class disease resistance protein	+	-	-	0.34	0.65	1.1
AT1G06180	MYB13	myb domain protein 13	-	+	-	0.34	0.57	2.18
AT4G29330	DER1	derlin-1	-	-	+	0.34	1.05	1.01
AT4G09460	MYB6	transcription repressor MYB6	-	+	-	0.33	0.37	1.15
AT1G32230	RCD1	inactive poly [ADP-ribose] polymerase RCD1	+	+	+	0.33	1.12	1
AT1G29340	PUB17	U-box domain-containing protein 17	+	-	-	0.33	1.1	0.95
AT5G07690	MYB29	myb domain protein 29	+	+	-	0.33	-0.91	-1.57
AT2G35940	BLH1	BEL1-like homeodomain 1	-	+	+	0.33	0.87	2.48
AT2G28200	AT2G28200	zinc finger protein ZAT5	-	-	+	0.33	-0.36	2.45
AT5G13680	ABO1	elongator complex protein 1	-	-	+	0.33	1.17	0.93
AT4G33920	APD5	putative protein phosphatase 2C 63	+	-	-	0.32	1.28	1.18
AT3G53260	PAL2	phenylalanine ammonia-lyase 2	+	-	+	0.32	-1.04	0.33
AT2G40000	HSPRO2	HS1 PRO-1 2-like protein	+	+	+	0.31	1.35	2.56
AT3G05020	ACP1	acyl carrier protein 1	+	-	-	0.3	-1.01	-0.95
AT5G59420	ORP3C	oxysterol binding protein-related protein 3C	+	+	+	0.29	1.07	0.95
AT5G47120	BI1	BAX inhibitor 1	+	+	-	0.29	1.29	1.58
AT3G05970	LACS6	long-chain acyl-CoA synthetase 6	+	-	-	0.29	1.55	1.79
AT5G07470	PMSR3	peptide methionine sulfoxide reductase A3	-	-	+	0.29	0.45	1.09
AT3G27890	NQR	NADPH:quinone oxidoreductase	+	+	+	0.28	0.63	1.29
AT5G20830	SUS1	sucrose synthase 1	-	+	+	0.28	2.26	1.42
AT1G66330	AT1G66330	putative senescence-associated protein	-	-	+	0.28	-1.31	1.2

AT5G52870	MAKR5	hypothetical protein	-	-	+	0.28	0.33	1.28
AT5G46180	DELTA-OAT	ornithine-delta-aminotransferase	+	-	-	0.28	0.3	1.18
AT1G23740	AOR	alkenal/one oxidoreductase	+	-	-	0.28	-1.76	0.27
AT3G03190	GSTF11	glutathione S-transferase F11	-	-	+	0.27	-0.44	-2.06
AT3G60190	DL1E	dynamin-related protein 1E	+	+	+	0.27	1.22	0.72
AT4G12480	EARLI1	putative lipid transfer protein	+	+	-	0.26	2.47	1.85
AT3G11280	AT3G11280	VHA-B1-interacting transcription factor, putative	-	+	-	0.26	0.65	1.9
AT5G39890	AT5G39890	hypothetical protein	-	+	+	A	2.06	A
AT5G18420	AT5G18420	hypothetical protein	-	-	+	0.25	1	0.64
AT3G08970	ATERDJ3A	DnaJ domain-containing protein	-	-	+	A	0.85	1.42
AT2G17520	IRE1A	endoribonuclease/protein kinase IRE1-like protein	+	+	-	0.22	1.12	1.28
AT4G22890	PGR5-LIKE A	PGR5-like protein 1A	+	-	-	0.21	-1.1	-0.88
AT3G21230	4CL5	4-coumarate--CoA ligase 5	+	-	-	0.21	1.57	1.36
AT1G10920	LOV1	disease susceptibility protein LOV1	+	-	-	A	0.8	1.14
AT5G67300	MYBR1	transcription factor MYB44	+	+	-	0.19	-0.4	1.4
AT3G50920	LPPepsilon1	phosphatidic acid phosphatase (PAP2) family protein	-	-	+	0.19	1.1	0.78
AT5G38895	AT5G38895	RING/U-box superfamily protein	-	-	+	0.18	1.2	1.14
AT3G57550	AGK2	guanylate kinase	+	-	-	0.18	0.56	1.09
AT2G21330	FBA1	fructose-bisphosphate aldolase 1	-	-	+	0.18	-1.64	-0.11
AT2G14580	PRB1	pathogenesis-related protein 1	-	+	-	0.18	-0.14	1.15
AT3G17410	AT3G17410	putative serine/threonine protein kinase	+	+	-	0.16	0.78	1.08
AT1G22180	AT1G22180	Sec14p-like phosphatidylinositol transfer family protein	+	-	-	0.16	1.64	1.51
AT2G31070	TCP10	transcription factor TCP10	+	+	+	0.16	-1.01	0.06
AT5G64930	CPR5	protein CPR-5	+	-	+	0.14	-0.28	1.29
AT2G30860	GSTF9	glutathione S-transferase PHI 9	+	-	-	0.14	0.26	-1
AT1G02170	MC1	metacaspase 1	+	+	-	0.14	1.05	0.9
AT5G65210	TGA1	transcription factor TGA1	+	+	-	0.12	0.58	1.57
AT5G61290	AT5G61290	flavin-containing monooxygenase FMO GS-OX-like 8	-	-	+	0.12	-0.04	-2.06
AT2G19310	AT2G19310	heat shock protein	-	-	+	0.12	1.34	0.77
AT3G24900	RLP39	receptor like protein 39	+	-	-	A	1.64	3.85
AT5G24590	TIP	TCV-interacting protein	+	-	-	0.11	0.83	1.04
AT5G58220	TTL	transthyretin-like protein	+	-	-	0.11	0.86	1.19
AT5G67160	EPS1	BAHD acyltransferase family protein	-	+	-	0.11	-0.12	2.23
AT2G29650	PHT4;1	sodium-dependent phosphate transport protein 1	+	+	+	0.11	-1.49	-0.93
AT4G29140	ADS1	putative MATE transport protein ADS1	+	-	-	A	1.2	A
AT1G18150	ATMPK8	mitogen-activated protein kinase 8	-	-	+	0.07	-0.17	-1.58
AT4G24670	TAR2	tryptophan aminotransferase related 2	+	-	-	0.06	-1.42	-0.99
AT5G22000	RHF2A	E3 ubiquitin-protein ligase RHF2A	-	+	-	0.06	-0.01	1.18
AT3G61250	MYB17	LATE MERISTEM IDENTITY2	-	+	-	0.06	-1.17	0.11
AT1G80130	AT1G80130	tetratricopeptide repeat domain-containing protein	-	-	+	0.06	-0.39	1.75
AT1G31540	AT1G31540	TIR-NBS-LRR class disease resistance protein	+	-	-	0.04	1.18	1.26
AT5G13220	JAZ10	protein TIFY 9	-	+	-	0.03	0.39	1.03
AT5G60970	TCP5	transcription factor TCP5	+	+	+	0.03	-1.25	-0.4
AT5G14930	SAG101	acyl hydrolase	+	+	-	0.02	1.42	1.92
AT4G12490	AT4G12490	Bifunctional inhibitor/lipid-transfer protein/seed storage 2S albumin superfamily protein	+	-	-	0.02	2.82	3.83
AT3G10800	BZIP28	putative bZIP transcription factor	+	+	-	0.01	1.02	1.27

AT5G58670	PLC1	phosphoinositide phospholipase C 1	+	+	+	0.01	-0.26	-1.53
AT1G02220	NAC003	NAC domain-containing protein	-	-	+	A	1.14	3.83
AT5G11920	cwINV6	beta-fructofuranosidase, insoluble isoenzyme CWINV6	-	+	+	A	1.33	3
AT5G60890	MYB34	myb domain protein 34	+	-	-	-0.01	-1.18	-0.36
AT3G55800	SBPASE	Sedoheptulose-1,7-bisphosphatase	+	+	+	-0.02	-1.16	-0.47
AT1G56600	GolS2	galactinol synthase 2	-	-	+	-0.03	-0.99	1.69
AT1G32220	AT1G32220	NAD(P)-binding Rossmann-fold-containing protein	-	-	+	-0.04	-1.04	-1.39
AT3G60980	AT3G60980	pentatricopeptide repeat-containing protein	-	-	+	-0.04	-0.51	-1.54
AT3G14470	AT3G14470	NB-ARC domain-containing disease resistance protein	+	+	-	-0.04	2.21	1.74
AT1G55210	AT1G55210	Disease resistance-responsive (dirigent-like protein) family protein	+	-	-	-0.04	0.83	1.63
AT5G10170	MIPS3	myo-inositol-1-phosphate synthase	-	-	+	-0.07	-0.03	-1.28
AT2G41430	ERD15	dehydration-induced protein ERD15	-	-	+	-0.07	-0.23	-1.29
AT5G02840	LCL1	protein LHY/CCA1-like 1	-	+	-	-0.08	-1.98	1.03
AT5G59780	MYB59	transcription factor MYB59	-	+	-	-0.11	-2.4	0.32
AT2G39730	RCA	ribulose bisphosphate carboxylase/oxygenase activase	+	+	+	-0.12	-1.02	-0.42
AT2G31180	MYB14	myb domain protein 14	-	+	-	-0.12	-2.41	-0.34
AT5G24320	AT5G24320	transducin/WD40 domain-containing protein	-	-	+	-0.12	-1.1	0.04
AT3G25020	RLP42	receptor like protein 42	+	-	-	-0.12	2.6	1.68
AT4G33150	AT4G33150	lysine-ketoglutarate reductase/saccharopine dehydrogenase bifunctional enzyme	-	-	+	-0.13	0.36	1.15
AT4G21980	APG8A	autophagy-related protein 8a	+	-	-	-0.14	0.5	1.23
AT3G29320	PHS1	alpha-glucan phosphorylase 1	+	-	-	-0.14	0.04	-3.23
AT1G78600	LZF1	light-regulated zinc finger protein 1	+	+	+	-0.14	-0.37	-3.41
AT3G08670	AT3G08670	hypothetical protein	-	-	+	-0.14	-1.02	-1.1
AT4G02195	SYP42	syntaxin-42	+	-	-	-0.16	0.44	1.17
AT1G15010	AT1G15010	hypothetical protein	+	-	-	-0.17	0.83	1.88
AT1G50290	AT1G50290	hypothetical protein	-	-	+	-0.17	-1.45	-0.05
AT3G17860	JAZ3	jasmonate-zim-domain protein 3	+	-	-	-0.17	-1.12	-0.67
AT5G37260	RVE2	MYB family transcription factor Circadian 1	-	+	-	A	2.04	1.68
AT2G26400	ARD3	acireductone dioxygenase 3	-	+	+	-0.18	4.7	5.08
AT3G15360	TRX-M4	thioredoxin M4	-	-	+	-0.18	-0.44	-1.09
AT5G47390	AT5G47390	myb-like transcription factor family protein	-	+	-	-0.19	-1.11	-0.68
AT4G35600	CST	putative serine/threonine-protein kinase Cx32	-	+	-	-0.19	1.48	2.52
AT3G54050	HCEF1	fructose-1,6-bisphosphatase	+	-	-	-0.19	-0.89	-1.1
AT5G37770	TCH2	calcium-binding protein CML24	-	-	+	-0.2	-0.31	1.23
AT2G13440	AT2G13440	glucose-inhibited division family A protein	-	-	+	-0.2	-0.47	-1.05
AT5G43750	PnsB5	NAD(P)H dehydrogenase 18	-	-	+	-0.21	-1.19	-1.08
AT1G54410	HIRD11	dehydrin family protein	-	-	+	-0.21	0.01	-1.21
AT1G73260	KTI1	kunitz trypsin inhibitor 1	+	+	+	A	A	5.54
AT4G29380	VPS15	phosphoinositide-3-kinase, regulatory subunit 4, p150	-	+	+	-0.21	0.73	1.16
AT2G33050	RLP26	receptor like protein 27///receptor like protein 26	+	-	-	-0.21	-0.34	-1.09
AT2G46220	AT2G46220	hypothetical protein	+	-	-	-0.22	-1.16	-0.56
AT3G05660	RLP33	receptor like protein 33	+	+	+	A	1.6	0.2
AT5G45410	AT5G45410	hypothetical protein	-	+	-	-0.23	-0.44	1.02
AT5G59080	AT5G59080	hypothetical protein	-	-	+	-0.23	-2.66	-2.16
AT2G23620	MES1	methyl esterase 1	+	-	-	-0.23	-1.42	0.12
AT1G32060	PRK	phosphoribulokinase	+	+	+	-0.24	-1.19	-0.74

AT1G21910	DREB26	ethylene-responsive transcription factor ERF012	-	+	-	-0.25	-1.01	-0.1
AT5G15120	AT5G15120	hypothetical protein	-	+	+	-0.26	1.23	-0.75
AT1G55870	AHG2	poly(A)-specific ribonuclease PARN	-	+	-	-0.28	-1.04	-0.05
AT2G22550	SVP	MADS-box protein SVP	+	+	+	-0.28	-1.06	-0.85
AT3G47620	TCP14	transcription factor TCP14	+	-	-	-0.28	-0.63	-1.74
AT3G14210	ESM1	epithiospecifier modifier 1	+	-	-	-0.29	-0.6	-1.26
AT3G52960	AT3G52960	peroxiredoxin-2E	+	-	-	-0.3	-0.41	-1.01
AT3G50820	PSBO2	oxygen-evolving enhancer protein 1-2	+	+	+	-0.3	-1.2	-0.85
AT4G35770	SEN1	senescence-associated protein DIN1	-	-	+	-0.31	-1.68	0.65
AT4G25100	FSD1	superoxide dismutase [Fe]	-	-	+	-0.32	-1.33	-0.58
AT2G02740	WHY3	single-stranded DNA-binding protein WHY3	+	-	-	-0.32	0.05	-1.16
AT2G14610	PR1	pathogenesis-related protein 1	+	+	+	-0.33	4.42	7.22
AT1G08810	MYB60	myb domain protein 60	-	+	-	-0.33	-0.44	-1.19
AT5G62790	DXR	1-deoxy-D-xylulose 5-phosphate reductoisomerase	-	-	+	-0.33	-1.17	-1.03
AT1G06080	ADS1	delta-9 acyl-lipid desaturase 1	+	-	-	-0.34	-1.03	-1.51
AT1G24240	AT1G24240	ribosomal protein L19 family protein	+	-	-	-0.35	-0.28	-1.33
AT5G04230	PAL3	phenylalanine ammonia-lyase 3	+	-	-	-0.35	-0.44	-1.27
AT1G75460	AT1G75460	ATP-dependent protease La domain-containing protein	+	+	+	-0.36	-1.51	-1.73
AT2G28800	ALB3	inner membrane protein ALBINO3	-	-	+	-0.37	-1.01	-0.63
AT2G32120	HSP70T-2	heat-shock protein 70T-2	-	-	+	-0.37	-1.09	-0.84
AT3G26932	DRB3	dsRNA-binding protein 3	+	-	-	-0.38	-1.21	-0.91
AT5G14740	CA2	carbonic anhydrase 2	+	+	+	-0.38	-1.11	-0.39
AT1G71030	MYBL2	putative myb family transcription factor	-	+	-	-0.4	-3.4	-2.98
AT3G47450	NOA1	NO-associated protein 1	-	-	+	-0.4	-0.51	-1.39
AT3G20820	AT3G20820	leucine-rich repeat-containing protein	+	-	-	-0.4	-1.26	-0.79
AT4G21870	AT4G21870	heat shock protein class V 15.4	-	-	+	-0.4	-2.22	-1.93
AT3G24590	PLSP1	plastidic type I signal peptidase 1	+	-	-	-0.4	-0.53	-1.08
AT4G35090	CAT2	catalase 2	-	-	+	-0.4	-3.44	0.96
AT4G37000	ACD2	red chlorophyll catabolite reductase	+	-	-	-0.4	-1.04	-1.4
AT1G13930	AT1G13930	salt tolerance-related protein	-	-	+	-0.42	-0.26	-3.48
AT5G27320	GID1C	putative gibberellin receptor GID1L3	-	+	-	-0.43	-1.32	-0.62
AT1G22640	MYB3	transcription factor MYB3	-	+	-	-0.43	-2.15	-0.66
AT3G01480	CYP38	peptidyl-prolyl cis-trans isomerase CYP38	+	+	+	-0.45	-0.81	-1.07
AT5G36910	THI2.2	thionin 2.2	+	-	-	-0.45	-2.2	-2.51
AT4G11660	AT-HSFB2B	heat stress transcription factor B-2b	-	-	+	-0.46	0.82	1.39
AT2G01570	RGA1	DELLA protein RGA	-	+	+	-0.46	-1.03	-0.03
AT5G45090	PP2-A7	phloem protein 2-A7	+	-	-	A	1.17	1.04
AT1G35680	RPL21C	50S ribosomal protein L21	+	-	-	-0.49	-0.87	-1.03
AT1G76080	CDSP32	thioredoxin-like protein CDSP32	-	-	+	-0.49	-1.18	-1.06
AT4G09650	ATPD	F-type H+-transporting ATPase subunit delta	+	+	+	-0.49	-1.5	-1.27
AT3G50700	IDD2	indeterminate-domain 2 protein	+	+	+	-0.51	-0.54	-2.98
AT3G16670	AT3G16670	pollen Ole e 1 allergen and extensin family protein	-	-	+	-0.51	-1.22	-1.5
AT3G15850	FAD5	palmitoyl-monogalactosyldiacylglycerol delta-7 desaturase	+	-	-	-0.51	-1.96	-0.74
AT4G16155	AT4G16155	dihydrolipoyl dehydrogenase	-	-	+	-0.51	-0.89	-1.33
AT3G54920	PMR6	putative pectate lyase	+	-	-	-0.51	-0.63	-1.39
AT1G20620	CAT3	catalase 3	+	-	+	-0.52	-0.4	-1.12

AT2G35690	ACX5	acyl-CoA oxidase 5	+	-	-	-0.52	-1.41	-0.72
AT5G52100	CRR1	dihydrodipicolinate reductase 3	+	-	-	-0.52	-0.93	-1.38
AT3G30775	ERD5	proline dehydrogenase 1	+	-	+	-0.52	-0.91	1.2
AT4G38840	AT4G38840	SAUR-like auxin-responsive protein	+	-	-	-0.55	-2.04	-0.63
AT1G60950	FED A	ferredoxin-2	+	-	-	-0.56	-1.16	-0.49
AT2G23960	AT2G23960	Class I glutamine amidotransferase-like superfamily protein	+	-	-	-0.57	-0.6	-1.83
AT1G51360	DABB1	dimeric A/B barrel domainS-protein 1	+	-	-	-0.57	-0.94	-1.17
AT5G63310	NDPK2	nucleoside diphosphate kinase II	-	-	+	-0.57	-0.71	-1.1
AT1G20020	FNR2	ferredoxin--NADP reductase, leaf isozyme 2	+	+	+	-0.6	-0.57	-2.19
AT1G03675	THM1	thioredoxin M1	+	-	+	-0.61	-0.83	-1.24
AT2G31360	ADS2	16:0delta9 desaturase 2	+	-	-	-0.61	-1.14	-3.4
AT1G20440	COR47	dehydrin COR47	+	-	-	-0.62	-0.33	-1.43
AT5G20630	GER3	germin-like protein subfamily 3 member 3	+	-	-	-0.63	0.3	-1.33
AT5G63780	SHA1	putative E3 ligase SHA1	+	+	+	-0.63	-1.44	-2.14
AT3G03450	RGL2	DELLA protein RGL2	-	+	+	-0.66	-0.78	1.02
AT1G76110	AT1G76110	high mobility group B protein 9	+	+	+	-0.66	-2.26	-0.78
AT1G02860	NLA	E3 ubiquitin-protein ligase BAH1	+	+	-	-0.68	-1.08	-1.03
AT4G39090	RD19	cysteine proteinase RD19a	+	-	-	-0.68	-0.28	-1.46
AT2G43010	PIF4	transcription factor PIF4	+	+	+	-0.69	-1.76	-2.47
AT5G51820	PGM	phosphoglucomutase	+	-	-	-0.7	-0.25	-1.27
AT1G68520	BBX14	zinc finger protein CONSTANS-LIKE 6	+	+	+	-0.72	-0.35	-2.35
AT5G08280	HEMC	Porphobilinogen deaminase	+	+	-	-0.73	-0.84	-1.02
AT3G03630	CS26	cysteine synthase 26	-	-	+	-0.73	-0.36	-1.18
AT5G62410	SMC2	structural maintenance of chromosome 2	+	-	-	-0.74	-0.19	-1.29
AT3G25010	RLP41	receptor like protein 41	+	-	-	-0.75	2.61	5.02
AT2G37220	AT2G37220	chloroplast RNA binding protein	+	+	-	-0.75	-0.49	-1.07
AT4G37260	MYB73	myb domain protein 73	-	+	-	-0.76	0.39	-1.12
AT5G17490	RGL3	DELLA protein RGL3	-	+	-	-0.77	-1.54	-3.7
AT5G15410	DND1	cyclic nucleotide-gated ion channel 2	+	-	-	-0.77	-1.41	-0.16
AT4G27450	AT4G27450	aluminum induced protein with YGL and LRDR motifs	-	+	+	-0.77	-0.99	1.97
AT2G44920	AT2G44920	thylakoid lumenal protein 1	+	-	-	-0.78	-1.45	-1.17
AT3G49870	ARLA1C	ADP-ribosylation factor-like A1C	+	-	-	-0.78	-1.38	-0.89
AT2G02100	LCR69	defensin-like protein 2	+	-	-	-0.79	0.51	-1.33
AT5G06290	2-Cys Prx I	2-Cysteine peroxiredoxin	+	+	+	-0.79	-0.65	-1.46
AT3G13750	BGAL1	beta galactosidase 1	-	-	+	-0.8	-1.37	-0.83
AT1G32540	LOL1	lsd one like 1 protein	+	-	-	-0.83	-2.09	-1.37
AT2G34600	JAZ7	jasmonate-zim-domain protein 7	+	-	+	A	A	4.42
AT2G48070	RPH1	resistance to phytophthora 1 protein	+	-	+	-0.86	-1.22	-1.27
AT1G09350	GolS3	galactinol synthase 3	-	-	+	-0.89	-2.55	-2.54
AT5G09290	AT5G09290	Inositol monophosphatase family protein	-	+	+	A	1	2.19
AT1G60870	MEE9	maternal effect embryo arrest 9 protein	-	-	+	-0.92	-1.14	0.04
AT4G38700	AT4G38700	disease resistance-responsive, dirigent domain-containing protein	+	-	-	-0.96	-1.31	-0.96
AT2G39010	PIP2E	plasma membrane intrinsic protein 2E	+	+	-	-0.96	-1.53	-0.68
AT4G30650	AT4G30650	putative low temperature and salt responsive protein	+	-	-	-0.98	0.41	-5.08
AT2G42530	COR15B	cold-regulated protein 15b	+	-	-	-0.99	-0.01	-4.99
AT2G21195	AT2G21195	hypothetical protein	-	-	+	-1.04	-2.03	-1

AT3G43250	AT3G43250	hypothetical protein	+	-	-	A	1.68	A
AT3G10020	AT3G10020	hypothetical protein	-	-	+	-1.05	-0.42	-2.74
AT1G79440	ALDH5F1	succinate-semialdehyde dehydrogenase	-	-	+	-1.05	0.83	-1.23
AT1G51090	AT1G51090	hypothetical protein	+	-	-	-1.06	-1.18	-3.02
AT2G37130	AT2G37130	peroxidase	+	-	-	-1.07	-0.13	0.91
AT2G44240	AT2G44240	hypothetical protein	-	-	+	A	A	2.2
AT3G48360	BT2	TAC1-mediated telomerase activation pathway protein BT2	-	+	+	A	2.25	A
AT3G56090	FER3	ferritin 3	-	-	+	-1.13	-0.25	0.09
AT5G08330	TCP11	transcription factor TCP21	+	+	+	-1.21	-1.63	-2.47
AT5G44920	AT5G44920	Toll-Interleukin-Resistance domain-containing protein	+	-	-	-1.24	-0.99	-1.05
AT1G64160	DIR5	dirigent-like protein DIR5	+	-	-	-1.25	-0.81	-1.27
AT4G00010	BRCA2(IV)	breast cancer protein 2 like 2A	+	-	-	-1.29	-1.04	-1.57
AT3G05800	AIF1	activation-tagged BRI1 suppressor 1-interacting factor 1	+	+	+	-1.32	-0.42	-1.36
AT4G01970	STS	stachyose synthase	-	-	+	-1.32	-1.03	-0.59
AT2G28820	PUB12	U-box E3 ubiquitin ligase	+	-	-	A	3.42	A
AT1G58290	HEMA1	glutamyl-tRNA reductase 1	-	-	+	-1.4	-3.01	-0.25
AT2G28900	OEP16-1	outer plastid envelope protein 16-1	+	-	-	-1.42	0.05	-3.14
AT2G15890	MEE14	maternal effect embryo arrest 14 protein	+	-	-	-1.43	-0.37	-4.96
AT1G49010	AT1G49010	myb/SANT-like DNA-binding domain-containing protein	-	+	-	-1.45	-1.28	0.04
AT3G47860	CHL	chloroplastic lipocalin	-	-	+	-1.47	-0.17	-2.25
AT4G20260	PCAP1	plasma-membrane associated cation-binding protein 1	+	-	-	-1.47	-1.26	-0.99
AT4G23310	CRK23	putative cysteine-rich receptor-like protein kinase 23	+	-	-	A	2.62	4.59
AT1G22770	GI	gigantea protein (GI)	-	-	+	-1.49	0.01	-3.68
AT2G40300	FER4	ferritin 4	-	-	+	-1.54	-1.72	0.65
AT4G13820	AT4G13820	leucine-rich repeat-containing protein	-	+	+	A	A	2.06
AT5G20250	DIN10	putative galactinol--sucrose galactosyltransferase 6	-	-	+	-1.57	-1.7	-1.56
AT5G14920	GASA14	gibberellin-regulated protein	-	-	+	-1.61	-0.1	-2.29
AT2G42360	AT2G42360	E3 ubiquitin-protein ligase ATL41	+	-	+	A	A	3.03
AT4G04770	ABCI8	ATP binding cassette protein 1	-	-	+	-1.66	-1.97	0.12
AT1G11210	AT1G11210	hypothetical protein	-	-	+	-1.76	0.19	-2.6
AT2G45760	BAP2	BON1-associated protein 2	-	+	-	A	5.47	4.55
AT1G20630	CAT1	catalase 1	-	-	+	-2.08	-0.74	-0.86
AT5G01600	FER1	ferretin 1	-	-	+	-2.58	-1.42	2.01
AT2G30480	AT2G30480	hypothetical protein	-	-	+	-2.84	-1.41	-1.06
AT5G51720	NEET	NEET group protein	-	-	+	-2.95	-4.03	0.11
AT4G04700	CPK27	calcium-dependent protein kinase 27	-	+	+	-3.13	0.17	-0.29
AT5G40990	GLIP1	GDSL lipase 1	+	+	-	A	A	1.08
AT1G09340	CRB	chloroplast stem-loop binding protein	+	+	+	A	A	A
AT4G02530	AT4G02530	chloroplast thylakoid lumen protein	+	+	-	A	A	A
AT3G23700	AT3G23700	ribosomal protein S1-like RNA-binding domain-containing protein	+	-	-	A	A	A
AT5G03940	CPSRP54	chloroplast signal recognition particle 54	+	-	-	A	A	A
AT3G04120	GAPC1	glyceraldehyde-3-phosphate dehydrogenase C subunit 1	-	-	+	A	A	A
AT3G01500	CA1	carbonic anhydrase 1	+	+	+	A	A	A
AT1G02200	CER1	protein ECERIFERUM 1	+	-	-	A	A	A
AT5G58260	NdhN	NADH dehydrogenase-like complex N	+	-	-	A	A	A
AT3G22400	LOX5	lipoxygenase 5	+	-	-	A	A	A

AT2G46600	AT2G46600	Calcium-binding EF-hand family protein	-	-	+	A	A	A
AT4G23670	AT4G23670	polyketide cyclase/dehydrase and lipid transport superfamily protein	+	-	-	A	A	A
AT1G14980	CPN10	chaperonin 10	-	-	+	A	A	A
AT5G47190	AT5G47190	50S ribosomal protein L19-2	+	-	-	A	A	A
AT1G42970	GAPB	glyceraldehyde-3-phosphate dehydrogenase B	+	-	+	A	A	A
AT3G23010	RLP36	receptor like protein 36	+	-	-	A	A	A
AT1G66100	AT1G66100	probable thionin-2.4	+	-	-	A	A	A
AT4G32190	AT4G32190	myosin heavy chain-related protein	-	-	+	A	A	A
AT3G15020	mMDH2	malate dehydrogenase 2	+	-	-	A	A	A
AT1G35580	CINV1	alkaline/neutral invertase CINV1	-	-	+	A	A	A
AT3G25070	RIN4	RPM1 interacting protein 4	+	-	-	A	A	A
AT1G59620	CW9	disease resistance (CC-NBS-LRR class) family protein	+	-	-	A	A	A
AT1G63350	AT1G63350	CC-NBS-LRR class disease resistance protein	+	-	-	A	A	A
AT4G17560	AT4G17560	50S ribosomal protein L19-1	+	-	-	A	A	A
AT3G59080	AT3G59080	aspartyl protease family protein	+	-	-	A	A	A
AT3G04290	LTL1	Li-tolerant lipase 1	-	+	-	A	A	A
AT1G11260	STP1	sugar transporter 1	+	-	-	A	A	A
AT2G02130	LCR68	defensin-like protein 1	+	-	-	A	A	A
AT2G45070	SEC61 BETA	protein transport protein sec61 subunit beta	-	-	+	A	A	A
AT2G33800	EMB3113	30S ribosomal protein S5	+	-	-	A	A	A
AT1G11860	AT1G11860	aminomethyltransferase	-	-	+	A	A	A
AT1G79380	RGLG4	ubiquitin ligase	+	-	-	A	A	A
AT2G26330	ER	LRR receptor-like serine/threonine-protein kinase ERECTA	+	-	-	A	A	A
AT2G35370	GDCH	glycine decarboxylase complex protein H	+	+	-	A	A	A
AT3G54560	HTA11	histone H2A 11	+	-	-	A	A	A
AT5G02480	AT5G02480	HSP20-like chaperone	-	-	+	A	A	A
AT1G69690	TCP15	transcription factor TCP15	+	+	+	A	A	A
AT3G50060	MYB77	myb domain protein 77	-	+	-	A	A	A
AT2G29450	GSTU5	glutathione S-transferase tau 5	-	-	+	A	A	A
AT3G49220	AT3G49220	putative pectinesterase/pectinesterase inhibitor 34	-	-	+	A	A	A
AT2G20570	GPRI1	transcription activator GLK1	+	+	+	A	A	A
AT4G14420	AT4G14420	HR-like lesion-inducing protein-like protein	-	-	+	A	A	A
AT4G18950	AT4G18950	Integrin-linked protein kinase family protein	-	+	-	A	A	A
AT3G16950	LPD1	lipoamide dehydrogenase 1	-	-	+	A	A	A
AT2G24150	HHP3	heptahelical protein 3	-	-	+	A	A	A
AT2G28190	CSD2	copper/zinc superoxide dismutase 2	-	-	+	A	A	A
AT3G19930	STP4	sugar transport protein 4	-	-	+	A	A	A
AT5G45800	MEE62	protein MATERNAL EFFECT EMBRYO ARREST 62	+	-	-	A	A	A
AT3G46130	MYB48	transcription factor MYB48	-	+	-	A	A	A
AT4G16860	RPP4	TIR-NBS-LRR class disease resistance protein	+	-	-	A	A	A
AT4G19530	AT4G19530	TIR-NBS-LRR class disease resistance protein	+	-	-	A	A	A
AT5G15310	MYB16	myb domain protein 16	-	+	-	A	A	A
AT3G26520	TIP2	aquaporin TIP1-2	+	-	+	A	A	A
AT2G41560	ACA4	autoinhibited Ca(2+)-ATPase 4	+	-	-	A	A	A
AT1G49430	LACS2	long-chain acyl-CoA synthetase 2	+	-	-	A	A	A
AT3G45290	MLO3	MLO-like protein 3	+	-	-	A	A	A

AT3G28460	AT3G28460	methyltransferase	+	-	-	A	A	A
AT1G54050	AT1G54050	CIII heat shock protein 17.4	-	-	+	A	A	A
AT5G18100	CSD3	copper/zinc superoxide dismutase 3	-	-	+	A	A	A
AT4G36030	ARO3	armadillo repeat only 3	+	+	-	A	A	A
AT3G07780	OBE1	protein OBERON 1	+	-	-	A	A	A
AT5G61420	MYB28	transcription factor MYB28	+	+	-	A	A	A
AT1G02205	CER1	protein ECERIFERUM 1	+	-	-	A	A	A
AT1G66350	RGL1	DELLA protein RGL1	-	+	+	A	A	A
AT5G50460	AT5G50460	protein transport protein sec61 subunit gamma-1	-	-	+	A	A	A
AT5G16000	NIK1	NSP-interacting kinase 1	+	-	-	A	A	A
AT3G11650	NHL2	NDR1/HIN1-Like protein 2	+	-	-	A	A	A
AT3G11630	AT3G11630	2-Cys peroxiredoxin BAS1	+	+	+	A	A	A
