# Supplementary material for: A Novel Pyrimidin-Like Plant Activator Stimulates Plant Disease Resistance and Promotes Growth
Source: PLoS One. 2015 Apr 7;10(4):e0123227. doi: 10.1371/journal.pone.0123227 (PMC4388471; doi:10.1371/journal.pone.0123227)
Supplement: S2 Table — (PDF) [file pone.0123227.s005.pdf]

S2 Table. Screen conditions of GO term

1. Defense:

GO:0052542: defense response by callose deposition
GO:0051607: defense response to virus
GO:0002213: defense response to insect
GO:0042742: defense response to bacterium
GO:0050832: defense response to fungus
GO:0006952: defense response
GO:0031347: regulation of defense response

2. ROS

GO:0042744: hydrogen peroxide catabolic process
GO:0010310: regulation of hydrogen peroxide metabolic process
GO:0042542: response to hydrogen peroxide
GO:0004784: superoxide dismutase activity
GO:0002679: respiratory burst involved in defense response
GO:2000377: regulation of reactive oxygen species metabolic process
GO:0034599: cellular response to oxidative stress
GO:0072593: reactive oxygen species metabolic process
GO:0000302: response to reactive oxygen species
GO:0050665: hydrogen peroxide biosynthetic process

3. Salicylic Acid

GO:0009863: salicylic acid mediated signaling pathway
GO:0052639: salicylic acid glucosyltransferase (ester-forming) activity
GO:0052640: salicylic acid glucosyltransferase (glucoside-forming) activity
GO:0080151: positive regulation of salicylic acid mediated signaling pathway
salicylic acid mediated signaling pathway
GO:2000031: regulation of salicylic acid mediated signaling pathway
regulation of salicylic acid biosynthetic process
salicylic acid mediated signaling pathway
GO:0009751: response to salicylic acid
regulation of salicylic acid metabolic process
GO:0046244: salicylic acid catabolic process
response to salicylic acid stimulus
GO:0010337: regulation of salicylic acid metabolic process
GO:0009697: salicylic acid biosynthetic process
GO:0080142: regulation of salicylic acid biosynthetic process
