# Supplementary material for: A Novel Pyrimidin-Like Plant Activator Stimulates Plant Disease Resistance and Promotes Growth
Source: PLoS One. 2015 Apr 7;10(4):e0123227. doi: 10.1371/journal.pone.0123227 (PMC4388471; doi:10.1371/journal.pone.0123227)
Supplement: S3 Table — (PDF) [file pone.0123227.s006.pdf]

S3 Table. Primers used in this study.

	Forward	Reverse
ACT2	GGTAACATTGTGCTCAGTGGTGG	GGTGCAACGACCTTAATCTTCAT
EDS1	TCTACGCTCAATGACCTT	CACCTGAATAATCTTCTTCTGAT
PAD4	TGACGCAATGGAGACTTA	TGATGAACAACAATGATACAGA
FMO1	CTGGTTATGATGGCAAGAAG	GCTCTGATGTGTGTAAGTTC
PBS3	GAATTAGCAGACACTATTGAAGA	CTCCGAAGAACCGTAAGT
WRKY40	TCTCACTATTGGCGTTACT	CTTCTTGTTCTCAGCACTAA
AGP5	TGTTGCTGTAGAGGCTATT	AAGAATGCCAATAATCAAGGAA
PR1	GTGAGGTGTAACAATGGTGGAA	CTTCATTAGTATGGCTTCTCGTTCA
PR2	GCTACAGAGATGGTGTCAGATTC	GTATCAGTGGTGGTGTCAGTG
PR5	CACAGACTTCACTCTAAGGAACA	AAGCACCTGGAGTCAATTCAA
ALD1	GCTACTCTTAGGCTCCAA	TCTGACCGATTATCACACTT
GST1	CCGTTTGTTTTTGGTTTATTCACTAAA	GATGAAAAGAAATACACAAATGGGAG
SID2	AGTTCTGTCTTCAACCACCTG	TCTATCTCCATATCACGAGCACTA
CYP71A13	GGGTAGAGGCTGGACCAAAT	ACAACCGAAGATGGAAATGC
PAD3	GGTACGGGATAAATCTCTATGA	AGATACAGTCGATGAACCTAC
PRX33	AATGTCCTCGCAATGGTAATCAA	ACTCTTGGTCGCTCTGGATAA
PRX34	CCTACACTCAACACTACTTACCT	CTCTTGGTCGCTCTGGATAA
APX1	GACTATCATCAACAGCGGCTAG	TGGCGAAGAGTGAAGTGAGT
WRKY30	ATCAGTTACCGAGGAATACAT	GTAGATGACAATGGATAGTGAAG

WRKY33	GAATGGTGGTGGAAGCAAGA	CTGTAACCGTCGTCAAGAATGT
RBOHD	GCCGAGCCGTATCTCCATTC	TCCAATGCCGAGACCTACGA
RBOHF	TCACAAATCAACGACGAGAGTT	CCCATCTTCATTCTTGTCCA
